# Structural Basis for the C-Terminal Domain of *Mycobacterium tuberculosis* Ribosome Maturation Factor RimM to Bind Ribosomal Protein S19

**DOI:** 10.3390/biom11040597

**Published:** 2021-04-18

**Authors:** Haoran Zhang, Qiuxiang Zhou, Chenyun Guo, Liubin Feng, Huilin Wang, Xinli Liao, Donghai Lin

**Affiliations:** MOE Key Laboratory of Spectrochemical Analysis & Instrumentation, Key Laboratory of Chemical Biology of Fujian Province, College of Chemistry and Chemical Engineering, Xiamen University, Xiamen 361005, China; hrzhang@outlook.com (H.Z.); Camille_xiang99@163.com (Q.Z.); guochy78@xmu.edu.cn (C.G.); tonyfeng@xmu.edu.cn (L.F.); wanghuilin@stu.xmu.edu.cn (H.W.); xlliao@xmu.edu.cn (X.L.)

**Keywords:** protein structure, *Mycobacterium tuberculosis*, ribosome maturation factor RimM, NMR spectroscopy, protein dynamics, MD simulation, protein–protein docking

## Abstract

Multidrug-resistant tuberculosis (TB) is a serious threat to public health, calling for the development of new anti-TB drugs. Chaperon protein RimM, involved in the assembly of ribosomal protein S19 into 30S ribosomal subunit during ribosome maturation, is a potential drug target for TB treatment. The C-terminal domain (CTD) of RimM is primarily responsible for binding S19. However, both the CTD structure of RimM from *Mycobacterium tuberculosis* (*Mtb*RimM_CTD_) and the molecular mechanisms underlying *Mtb*RimM_CTD_ binding S19 remain elusive. Here, we report the solution structure, dynamics features of *Mtb*RimM_CTD_, and its interaction with S19. *Mtb*RimM_CTD_ has a rigid hydrophobic core comprised of a relatively conservative six-strand β-barrel, tailed with a short α-helix and interspersed with flexible loops. Using several biophysical techniques including surface plasmon resonance (SPR) affinity assays, nuclear magnetic resonance (NMR) assays, and molecular docking, we established a structural model of the *Mtb*RimM_CTD_–S19 complex and indicated that the β4-β5 loop and two nonconserved key residues (D105 and H129) significantly contributed to the unique pattern of *Mtb*RimM_CTD_ binding S19, which might be implicated in a form of orthogonality for species-dependent RimM–S19 interaction. Our study provides the structural basis for *Mtb*RimM_CTD_ binding S19 and is beneficial to the further exploration of *Mtb*RimM as a potential target for the development of new anti-TB drugs.

## 1. Introduction

As a deadly infectious disease, tuberculosis (TB) infected about 10 million people and caused an estimated 1.4 million deaths worldwide in 2019, and the responsible pathogen for TB is *Mycobacterium tuberculosis* (*Mtb*) [1]. Globally, TB has developed resistance to traditional anti-TB drugs like isoniazid and rifampicin, an unfortunate complication to TB prevention and treatment [2,3]. Patients infected with multidrug-resistant TB (MDR-TB) require medicines at higher costs and a longer time for treatment, only receiving a treatment success rate of 57% [1]. With the emergence and spread of MDR-TB strains, it is imminent to find clinical targets for developing new antimicrobials against *Mtb*.

RimM, an important ribosome maturation factor protein existing in *Mtb*, is a candidate target for anti-TB drugs. The RimM protein family is included in various bacterial species, but no ortholog is available in humans. Researches have been extensively conducted on explicit functions of RimM serving as one of bacterial biogenesis factors active in the ribosome assembly process [4]. Knockout of the RimM gene caused a significant decrease in cell growth rate, accumulation of 16S rRNA precursors and ribosomal intermediates, and a reduction of polysome level [5,6,7,8]. Further researches showed that RimM does not bind to complete 70S ribosomes or mature 30S subunits but moderately binds to immature 30S intermediates [4,5,9]. Suppressive mutation experiments on ribosomal proteins and 16S rRNA indicated that RimM might bind the 3′-domain of 16S rRNA [5,8]. Analyses on composition and structures of the immature 30S intermediates collected in the RimM knockout strain indicated that RimM plays a crucial role in assisting the late assembly of the head domain of the 30S subunit [4,10,11]. Moreover, in vitro pull-down experiments confirmed the specificity with which RimM binds to S19, a ribosomal protein located at the 30S head domain [8]. Kinetic experiments disclosed that RimM accelerates the binding of S19 to 16S rRNA by overcoming the potential well during the slow binding process [12]. Together, these advances indicate that the RimM–S19 interaction plays a vital role in ribosome biosynthesis, for which RimM is a competent attacking target for antimicrobials against *Mtb*.

**Figure 1:**
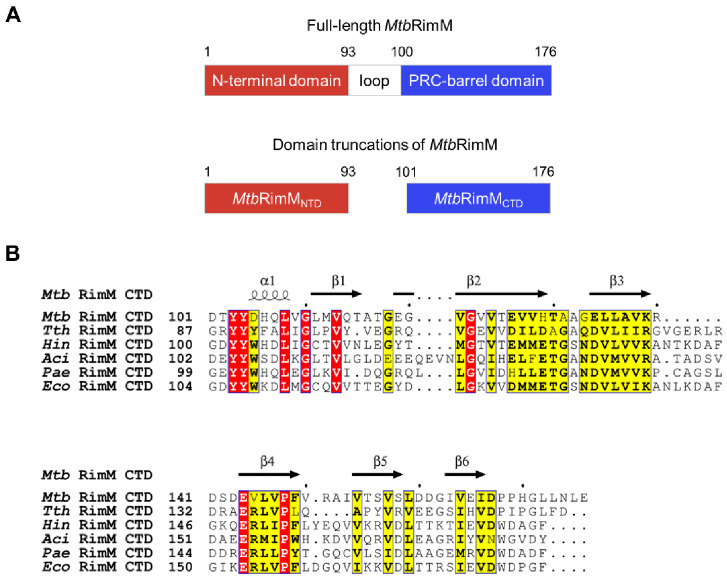
RimM consists of the N-terminal domain (NTD) and C-terminal domain (CTD). (**A**) Schematic diagram of two domains in RimM from *Mycobacterium Tuberculosis* (*Mtb*RimM) predicted by Pfam. (**B**) Sequence alignments between CTDs of *Mtb*RimM and RimM orthologs. Identical residues are highlighted in red and similar residues in yellow, and secondary structure elements of the CTD of *Mtb*RimM (*Mtb*RimM_CTD_) resolved in this study (see Section 3.1) are shown above the alignments.

As is well known, the function of a protein is determined by its structure, and structural basis is required to mechanistically understand the protein function. So far, the three-dimensional (3D) structure of RimM from *Mtb* (*Mtb*RimM) has not been determined. To our knowledge, the following 3D structures of RimM orthologs have been resolved and are accessible in the Protein Data Bank (PDB): (a) the crystal structures of RimM proteins in free form from *P. aeruginosa* (3 mutations, PDB ID: 2F1L, termed *Pae*RimM), *T. thermophilus* HB8 (wild type, PDB ID: 2DYI, termed *Tth*RimM), *A. calcoaceticus* (5 mutations, PDB ID: 2QGG, termed *Aci*RimM), and *H. influenzae* (wild type, PDB ID: 3H9N, termed *Hin*RimM); (b) the crystal structure of the RimM–S19 complex from *T. thermophilus* HB8 (both wild types, PDB ID: 3A1P, S19 from *T. thermophilus* is termed *Tth*S19); (c) the solution structure of truncated RimM N-terminal domain from *T. thermophilus* HB8 (wild type, PDB ID: 2DOG) [13]. Inspection of these structures, in correspondence to earlier assertions based on multi-sequence alignments [8,14], shows that the structure of RimM is composed of an N-terminal domain (NTD), a C-terminal domain (CTD), and a short loop in between (Figure 1A). Part of the RimM NTD shares sequence similarity with the RNA-binding KH domain, implying its potential interaction with RNA [5,15]. The RimM CTD was identified as a PRC-barrel domain [14]. Originally, the PRC-barrels were discovered to be a mediator of quinone reduction within the photosynthetic reaction center complex from purple proteobacteria [16]. However, PRC-barrels of the RimM family lack a decisive glutamate residue required for electron transfer in the redox reaction [14]. Instead, they are likely to be involved in binding S19 during the maturation of the 30S ribosomal subunit [8]. Previous nuclear magnetic resonance (NMR) titration assays showed that the CTD part of full-length RimM can significantly interact with S19 but the NTD part can not, indicating that the CTD part is primarily responsible for binding S19 [13].

Suffering from very few available conformational restraints, the structural calculation of *Tth*RimM CTD failed to converge in the NMR structure ensemble, in sharp contrast with *Tth*RimM NTD [13]. On the other hand, the aforementioned crystal structures of full-length RimM orthologs characterize a well-folded CTD, and the *Tth*RimM–S19 complex even describes a molecular model for the RimM–S19 interaction. However, sequence alignments show that the highest sequence identity between *Mtb*RimM CTD (termed *Mtb*RimM_CTD_) and other CTDs from species with known structures is only 32.48% (Figure 1B). Thus, the orthologous structures of RimM are insufficient for revealing the structural basis of *Mtb*RimM_CTD_ binding S19. Expectedly, such a structural basis would facilitate the design of RimM-based anti-TB drugs.

Here, we determined the solution structure of *Mtb*RimM_CTD_ that is primarily responsible for the interaction of *Mtb*RimM with S19. We then analyzed dynamics features of *Mtb*RimM_CTD_ by NMR relaxation measurements and molecular dynamics (MD) simulation, and characterized biophysical properties of *Mtb*RimM_CTD_ binding S19 through NMR titration and surface plasmon resonance (SPR) assays. Based on the identified S19 binding sites on *Mtb*RimM_CTD_*,* which were verified by mutagenesis experiments, we established a structural model of the *Mtb*RimM_CTD_–S19 complex by molecular docking to illustrate the unique pattern of *Mtb*RimM_CTD_ binding S19. Our results shed light on the molecular mechanisms of the *Mtb*RimM–S19 interaction and offer novel insights into drug development against tuberculosis.

## 2. Materials and Methods

### 2.1. Cloning, Expression, and Purification

The gene sequences of RimM and S19 from *M. tuberculosis* strain H37Rv (*Mtb*RimM and *Mtb*S19) were obtained from the NCBI database (gene ID: 887188 for RimM and 888356 for S19). Recombinant plasmids harboring *Mtb*RimM gene (pET-22b, with a C-terminal His_6_-tag) or *Mtb*S19 gene (pET-28a, with thrombin-cleavable N-terminal His_6_-tag) were commercially synthesized (GenScript, Nanjing, China) Boundaries for the N-terminal domain (residues 4–93, NTD) and the C-terminal domain (residues 100–173, CTD) of *Mtb*RimM were determined by Pfam [17]. To obtain truncations, individual gene fragments of the NTD of *Mtb*RimM (*Mtb*RimM_NTD_, residues 1–93) and the CTD of *Mtb*RimM (*Mtb*RimM_CTD_, residues 101–176) were extracted and cloned by PCR. The following primers were applied in the PCR: (a) 5′-TTAGGATCCATGGAGCTGGTTGTGG-3′ (restriction site BamH I) and (b) 5′-TAGCTCGAGTTAATCGTCCGCATCG-3′ (restriction site Xho I) for *Mtb*RimM_NTD_, and (c) 5′-CGCCGGCATATGGATACCTACTATG-3′ (restriction site Nde I) and (d) 5′-TAGCTCGAGTTCCAGGTTCAGCAGA-3′ (restriction site Xho I) for *Mtb*RimM_CTD_. PCR products for *Mtb*RimM_NTD_ were then ligated into the pET-28a-SUMO plasmid which contains an N-terminal His_6_-tag followed by a SUMO fusion protein and a SUMO protease cutting site [18], and those for *Mtb*RimM_CTD_ into the pET-22b plasmid which contains a C-terminal His_6_-tag. Recombinant plasmids encoding *Mtb*RimM_CTD_ point mutants (pET-22b, with C-terminal His_6_-tag) were commercially synthesized (Sangon Biotech, Shanghai, China). All plasmids used in this study were verified via DNA sequencing.

The plasmids bearing the genes of *Mtb*RimM, *Mtb*S19, *Mtb*RimM_NTD_, *Mtb*RimM_CTD_ and point mutants were transformed into the *E. Coli* BL21(DE3) strain. Overexpression of these proteins was induced with 0.5 mM IPTG at OD_600_ = 0.6 and conducted at 25 °C for 10 h in either LB liquid media or M9 media. For uniform ^15^N- and/or ^13^C-labeling of the proteins, 0.1% (*m*/*v*) of ^15^NH_4_Cl and/or 0.3% (*m*/*v*) of ^13^C-glucose were added into M9 media.

The harvested cell pellet was resuspended in 50 mM Tris, pH 8.0, 950 mM NaCl, 2.0 mM imidazole, 0.1 mg/mL lysozyme, 1.0 mM phenylmethylsulfonyl fluoride (PMSF), and lysed on ice by sonication. The soluble fraction of the lysate was collected by centrifugation and loaded onto 5 mL Co-NTA resin, which was obtained by stripping the Ni-NTA resin (GE Healthcare Bio-Sciences AB, Uppsala, Sweden) of coordinated Ni^2+^ with EDTA and re-cobaltizing it with CoCl_2_. In the affinity chromatography, recombinant protein was eluted with 50 mM Tris, pH 8.0, 250 mM NaCl, 300 mM imidazole. After that, the protein was buffer-exchanged into 20 mM potassium phosphate, pH 7.2, 100 mM KCl, 0.02% NaN_3_ (hereafter referred to as the universal buffer), and purified through size exclusion chromatography (SEC) using ÄKTA FPLC system with a Superdex 75 10/300 GL column (GE healthcare). Additionally, proteins with cleavable N-terminal His_6_-tags were treated with corresponding proteases until the tags were completely cleaved before repurified with Co-NTA and SEC. *Mtb*RimM_NTD_ was cleaved with 0.4 mg/mL SUMO-protease for 3 h at room temperature, and *Mtb*S19 with 0.4 mg/mL thrombin overnight at 4 °C. Finally, the protein solution was concentrated to 600 μM for NMR experiments conducted in this study if not otherwise specified.

### 2.2. NMR Spectroscopy

NMR spectra were recorded at 298K on a Bruker Avance III 850 MHz spectrometer (Bruker BioSpin GmbH, Karlsruhe, Germany) equipped with a ^1^H-/^13^C-/^15^N- TCI cryogenic probe (Bruker AG, Fällanden, Switzerland). All protein samples for NMR spectroscopy were dissolved in the universal buffer. The protein samples included 10% D_2_O (*v*/*v*) for recording general NMR spectra and 99% D_2_O (*v*/*v*) for recording 3D ^13^C-edited NOESY-HSQC (nuclear Overhauser effect spectroscopy-heteronuclear singular quantum correlation) spectrum. The [^15^N]-labeled protein sample was used for recording two-dimensional (2D) ^1^H-^15^N HSQC and 3D ^15^N-edited NOESY-HSQC spectra, and the [^13^C, ^15^N]-labeled protein sample was prepared for 2D ^1^H-^13^C HSQC and other 3D NMR spectra. 2D ^1^H-^15^N HSQC and ^1^H-^13^C HSQC spectra were recorded on *Mtb*RimM, *Mtb*RimM_NTD_ and *Mtb*RimM_CTD_. 3D HNCACB, CBCA(CO)NH, HNCA, HN(CO)CA, HNCO and HN(CA)CO spectra were recorded for performing backbone resonance assignments of *Mtb*RimM and *Mtb*RimM_CTD_. 3D H(CCO)NH, CC(CO)NH, HBHA(CO)NH, H(C)CH-COSY, and (H)CCH-TOCSY spectra were recorded for conducting side-chain resonance assignments of *Mtb*RimM_CTD_. 3D ^13^C-edited NOESY-HSQC and ^15^N-edited NOESY-HSQC spectra with a mixing time of 120 ms were recorded on *Mtb*RimM_CTD_ for obtaining NOE (nuclear Overhauser effect) restraints. All spectra were processed with NMRPipe [19] and analyzed with NMRFAM-SPARKY [20].

### 2.3. Structure Determination

Chemical shifts of *Mtb*RimM_CTD_ which had been previously deposited to BMRB (Accession ID: 36368) were used for NOE assignments (data under review) [21]. Cross-peaks in ^13^C- and ^15^N-edited NOESY-HSQC spectra were either manually assigned or ambiguously assigned with the help of Aria 2.3 [22]. NOE-derived distance restraints were generated from signal integrals in ^13^C- and ^15^N-edited NOESY-HSQC spectra. Backbone dihedral restraints (φ, ψ) were predicted from chemical shifts via the TALOS-N server [23]. Altered MD parameters were adopted in Aria 2.3 setup (10,000, 4000, 80,000, and 64,000 for high temperature, refinement, cool1, and cool2 steps). Totally, 100 structures of *Mtb*RimM_CTD_ were calculated and refined with Aria 2.3, and 20 lowest-energy models were used as the ultimate structure ensemble. The C-terminal His_6_-tag was not modeled in the final structures due to the lack of chemical shifts and NOE cross-peak assignments. PROCHECK [24] was applied for structural quality evaluation, and Pymol [25] for structure visualization.

### 2.4. NMR Relaxation Measurements

All recombinant proteins were dissolved in the universal buffer. A protein sample of [^15^N]-labeled *Mtb*RimM_CTD_ at 600 μM was used to conduct NMR relaxation measurements of backbone amide groups including amide R_1_ and R_2_ relaxation rates and {^1^H}-^15^N heteronuclear steady-state NOEs (hNOEs). Two sets of pseudo-3D experiments incorporated with ^1^H-^15^N HSQC spectra were recorded at 298K, 850 MHz for R_1_ and R_2_ measurements, and repeated 2D hNOE spectra were recorded at the same condition for hNOE measurements and error analysis. R_1_ values were calculated with relaxation delays of 10, 50, 100 (×2), 200, 400, 600, 800 (×2), 1200, 1600, and 2000 ms, while R_2_ values were determined with relaxation delays of 16.32, 32.64 (×2), 48.96, 65.28, 81.60, 97.92, 114.24, 130.56 (×2), 146.88, and 163.20 ms. The hNOEs were obtained in interleaved spectra with and without a 3-s ^1^H pre-saturation, the latter being replaced by a 3-s relaxation delay. Peak intensities were represented by peak heights for data analysis. NMRFAM-SPARKY [20] was used to fit exponential decay curves to the experimental serial data for determining R_1_ and R_2_ rates, where standard errors of rate constants were estimated by the spread in five repeated Gaussian distribution fits for random noise perturbing peak heights. Residues 131, 151, and 154 were subjugated to signal overlapping or broadening, thus unavailable for spin relaxation analysis. Therefore, a total of 71 residues were analyzed to access backbone dynamics, with prolines, the starting methionine, and the C-terminal His_6_-tag excluded.

### 2.5. Model-Free Analysis

The FAST-Modelfree program (Version 1.3, Loria Lab, New Haven, CT, USA) [26] was applied to extract dynamics parameters (S^2^, τ_e_, R_ex_) based on relaxation data of *Mtb*RimM_CTD__,_ which used estimated initial tensors of the protein as input. The Tensor 2 program was employed to estimate the initial tensors [27]. Spin diffusion tensors were then iteratively optimized under an axially-symmetric model. The closest-to-average model in the *Mtb*RimM_CTD_ structure ensemble (hereafter referred to as the *Mtb*RimM_CTD_ representative structure) determined in this work was prepared as the structure input for both Tensor 2 and FAST-Modelfree automation. The values of grid-search steps and convergence limit in the FAST-Modelfree setup were 15 and 0.001 for each tensor, respectively. The S^2^ cutoff was set to 0.4 for an all-encompassing characterization of residue spins.

### 2.6. NMR Titration Assays

All recombinant proteins were dissolved in the universal buffer. Either 500 μM [^15^N]-labeled *Mtb*RimM_NTD_ or 290 μM [^15^N]-labeled *Mtb*RimM_CTD_ was titrated with unlabeled *Mtb*S19 to a respectively equimolar ratio. The titration of *Mtb*RimM_CTD_ into *Mtb*S19 yielded precipitation, a factor unfavorable for a titration assay with higher protein concentrations. For mutual equimolar titrations between *Mtb*RimM_NTD_ and *Mtb*RimM_CTD_, both proteins at a concentration of 150 μM were used. ^1^H-^15^N HSQC spectra were recorded at each titration point at 298K. The chemical shift perturbation (CSP) was determined with an empirical formula [28] as

(1) $$\Delta \delta =\sqrt{\frac{1}{2}[{\left(\Delta \delta_{H}\right)}^{2}+0.14{\left(\Delta \delta_{N}\right)}^{2}]}$$

in which Δ*δ_H_* and Δ*δ_N_* represented chemical shift displacements for ^1^H and ^15^N nuclei observed upon titrations, respectively.

### 2.7. Sequence Alignments

Structure-based sequence alignments among the CTDs of RimM orthologs or among S19 orthologs were generated by Clustal X2 [29]. Visualization of the alignments was performed with ESPript 3.0 [30], where the secondary structure information was extracted by the STRIDE webserver using the determined PDB coordinates of *Mtb*RimM_CTD_ [31].

### 2.8. MD Simulation

Molecular dynamics simulation was executed under the ff19SB force field [32] integrated into the AmberTools20 suite [33]. The representative structure of *Mtb*RimM_CTD_ was cleaned up with the *pdb4amber* subroutine before the protein was solvated in an OPC water box [34] extending 10.0 Å from the protein surface. Ten Na^+^ ions were added to the system to neutralize the net charge of *Mtb*RimM_CTD_. A two-stage energy minimization was performed to discard bad contacts: first, the water molecules alone; next, the entire system. After that, a three-stage system equilibration totaling 300 ps was conducted: the system was heated from 0 to 300 K under NVT ensemble for 100 ps and was run at 300 K under NVT and NPT ensembles successively for a respective 100 ps. Subsequently, a 120-ns MD simulation under NPT ensemble at 300 K was performed. Langevin dynamics was adopted for temperature regulation with a collision frequency of 1 ps^−1^ and a time-based pseudo-random seed. Hydrogen-involving bonds were constrained by the SHAKE algorithm [35] and omitted for force evaluation. The nonbonded cutoff was specified as 12 Å.

The *cpptraj* subroutine [36] incorporated within AmberTools20 was utilized for MD data extraction and analysis. Root mean square fluctuation (RMSF) per residue throughout the simulation was calculated with reference to the representative structure of *Mtb*RimM_CTD_, to which the structures in all frames were RMS-fitted prior to calculation. Secondary structures were determined using the built-in DSSP engine [37].

### 2.9. Molecular Docking

Due to possible backbone conformational changes upon *Mtb*RimM_CTD_–S19 binding, molecular docking with backbone flexibility [38] was carried out via RosettaDock 4.0 (Rosetta Commons) [39]. As the two starting structures, the representative structure of *Mtb*RimM_CTD_ (termed dA) was chosen, and the structural model of *Mtb*S19 (termed dB) was built by homology modeling via the SWISS-MODEL server [40], using the crystal structure of S19 in RimM-complexed form from *T. thermophilus* HB8 (PDB: 3A1P) as the modeling template. The binding interface identified from chemical shift mapping described in this work was considered to engender a rough initial model (termed dC) containing both dA and dB. Totally, 100 conformational ensembles of each protein (termed dA′ and dB′, respectively) for backbone-flexible docking were generated using dA and dB under the unconstrained *relax* protocol. dC was also *relax*ed to spawn a clash-relieved model (termed dC′) after local refinement. Then, the *prepack* protocol was run to optimize side-chain rotamers. Finally, with dA+dA′ and dB+dB′ as ensemble candidates for backbone switch and dC′ as the initial input structure, unconstrained backbone-flexible docking of *Mtb*RimM_CTD_–S19 was performed and 30,000 docking models were calculated. The docking results were evaluated based on the RosettaDock interface energy score. In addition, the method developed by Kumar et al. can be applied to select a particular model out of a large number of docked models, which uses certain constraints for the docking and is then based on the lowest energy plot [41]. Random perturbation subjugated to Gaussian distribution was applied to the input structure prior to every individual simulation, with standard deviations of 3 Å for translation and 8° for rotation.

### 2.10. SPR Affinity Assays

All recombinant proteins were dissolved in 20 mM potassium phosphate, pH 7.2, 100 mM KCl (also used as the system running buffer) for SPR affinity assays. All experiments were performed at 298 K on a Biacore T200 instrument (GE Healthcare Bio-Sciences AB, Uppsala, Sweden). The sandwich approach was employed for the SPR assays: first, the anti-histidine antibody from the His Capture Kit (Cytiva Sweden AB, Uppsala, Sweden) was immobilized to the active surface of a CM5 sensor chip (Cytiva Sweden AB, Uppsala, Sweden) until saturation; second, excessive C-terminal His_6_-tagged *Mtb*RimM_CTD_ or its mutants flowed through and were captured by the anti-histidine antibody; last, a serial concentration of *Mtb*S19 was injected into the system and captured by *Mtb*RimM_CTD_. The control surface was treated in the same way, except that *Mtb*S19 solution was substituted by blank running buffer. The following concentrations of *Mtb*S19 were used for obtaining SPR assay curves: 0, 0.25, 0.50, 1.0, 2.0, 4.0, and 8.0 μM. Dissociation constants (K_D_) were determined by fitting the SPR assay curves to the steady-state model.

## 3. Results

### 3.1. Solution Structure of MtbRimM_CTD_

We first determined the sequential boundaries of NTD and CTD within *Mtb*RimM for the structural determination of *Mtb*RimM_CTD_. The prediction using Pfam [17] showed that the NTD of *Mtb*RimM covered residues 4–93, the CTD spanned residues 100–173, and in between lay a flexible loop as a linker (Figure 1A). Considering that residue P100 is nonconserved and too hydrophobic as a terminal residue, we omitted P100 in our CTD truncation design to maintain protein solubility. Consequently, we designed recombinant truncations for the NTD (residues 1–93, termed *Mtb*RimM_NTD_) and the CTD (residues 101–176, termed *Mtb*RimM_CTD_), and prepared protein samples for further studies.

**Figure 2:**
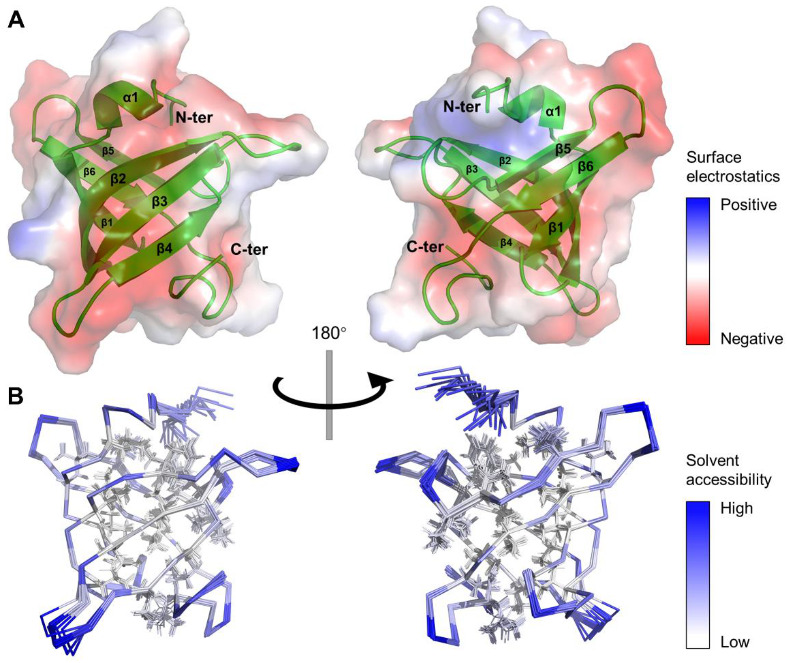
Three-dimensional structure of *Mtb*RimM_CTD_ in solution. (**A**) Cartoon depiction and surface electrostatics display for the mean structure of *Mtb*RimM_CTD_. Positive and negative charges are colored in blue and red on the protein surface with 50% transparency, respectively. (**B**) Ribbon depiction of 20 lowest-energy models for *Mtb*RimM_CTD_. Relative solvent accessibility per residue is colored from white to blue in ascending order. Particularly, side chains of non-polar residues buried in the hydrophobic core are presented as lines.

Chemical shifts for *Mtb*RimM_CTD_ with 99% backbone ^15^N-^1^H resonances and 85% side-chain aliphatic ^1^H/^13^C resonances assigned were previously deposited to BMRB (accession ID: 36368), and ^13^C-edited and ^15^N-edited NOESY-HSQC spectra were also recorded (under review) [21]. Based on these data, we obtained NOE distance restraints and predicted dihedral angle restraints to calculate the solution structure of *Mtb*RimM_CTD_. We determined a *Mtb*RimM_CTD_ ensemble of 20 lowest-energy models and submitted it to Protein Data Bank (PDB ID: 7CQ1). The NMR restraints and structural statistics are listed in Table 1. The root-mean-squared deviation (RMSD) of backbone atomic coordinates of the ordered region (residues 103–173) to the mean structure reached 0.23 Å, indicative of a well-defined structure ensemble. *Mtb*RimM_CTD_ adopted a β-barrel structure containing a short α-helix (α1:D105-L108) and a β-sheet composed of six strands (β1: L111-T115, β2: E119-H129, β3:E134-K139,β4:E144-F149,β5:V154-S158,β6:I163-I166) in both anti-parallel (β1-β2-β3-β4, β5-β6) and parallel orientations (β6-β1), spatially arranged in the order of β5-β6-β1-β2-β3-β4 (Figure 2A). The short α1 helix was situated on the top of the β-barrel.

It is noteworthy that most residues on the β-strands with side chains facing inward were hydrophobic residues, thus forming a hydrophobic core (Figure 2B). Meanwhile, side chains of most charged or polar residues were oriented toward the outer side of the β-barrel, permitting direct polar contacts to water molecules. The dispersion of positive surface charges was somewhat scattered, while negatively charged residues clustered into larger blocks (Figure 2A). Considering the role of CTD as the S19 binder, these extended negatively charged blocks potentially enabled the binding of positively charged ligands/residues. Non-polar residues that formed the hydrophobic core shared high similarities among species (Figure 1B), implying the need for a stabilizing engine for RimM CTD in evolution. In contrast, the low sequence conservation of surface residues might result in functional distinctions between *Mtb*RimM_CTD_ and its orthologs.

### 3.2. Structural Comparisons between MtbRimM_CTD_ and Its Orthologs

**Figure 3:**
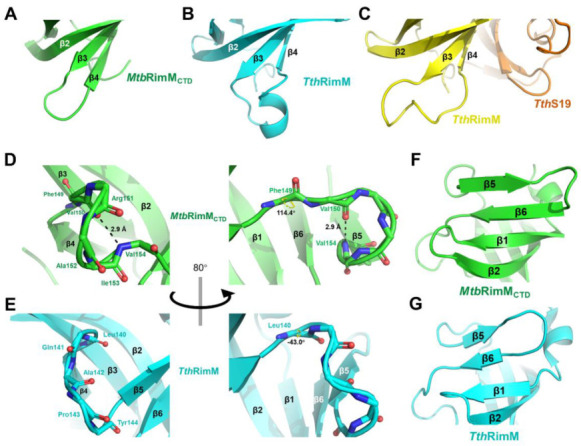
Structural comparison between *Mtb*RimM_CTD_ and RimM from *T. thermophilus* HB8 (*Tth*RimM) represented in cartoon. (**A**–**C**) β3-β4 loop of *Mtb*RimM_CTD_ (**A**), free *Tth*RimM (**B**), S19-complexed *Tth*RimM (**C**). (**D**,**E**) β4-β5 loop of *Mtb*RimM_CTD_ (**D**) and free *Tth*RimM (**E**). The dihedral angle ψ of F149 in (**D**) and L140 in (**E**) are identified. The length of the hydrogen bond (V154)N-H…O(V150) is also depicted in (**D**), where backbone oxygen or nitrogen atoms are shown as red and blue sticks, respectively. Hydrogen atoms, if applicable, are hidden. (**F**,**G**) β5 and β6 strands of *Mtb*RimM_CTD_ (**F**) and free *Tth*RimM (**G**).

To investigate structural distinctions between *Mtb*RimM_CTD_ and its orthologs, we performed CTD-centered structural superpositions based on sequence alignments. Surprisingly, coordinate RMSD was no more than 1.992 Å for the orthologs despite low sequence identity, showing a high degree of structural similarity among the CTDs of RimM (Figure S1). The CTD of *Tth*RimM, the one with the largest RMSD to *Mtb*RimM_CTD_, displayed notable structural distinctions in the β3-β4 loop. In *Mtb*RimM_CTD_, the shorter β3-β4 loop showed less flexibility and took on a turn-like conformation (Figure 3A). In *Tth*RimM, residues R129–R133 in this loop form a short 3_10_-helix (Figure 3B). By contrast, the S19-complexed *Tth*RimM showed a reduced turn-like loop instead of a helix (Figure 3C), possibly hampered by spatial hindrance (Q56 of *Tth*S19 to E135 of *Tth*RimM) or electrostatic repulsion (K32 of *Tth*S19 to R131 of *Tth*RimM). Given that the β3-β4 loop in *Tth*RimM underwent significant conformational change once the protein binding S19, whether this loop in *Mtb*RimM_CTD_ showed a similar pattern is worthy of examination.

On the other hand, the β4-β5 loop in *Mtb*RimM_CTD_ consisted of a helix-like fold, characterized by the hydrogen bond between V150 and V154 (Figure 3D). This hydrogen bond also exists among the RimM orthologs with known structures, except for *Tth*RimM. In fact, despite the backbone N-C_α_-C atoms of L140 in *Tth*RimM adopted a similar orientation to the corresponding F149 in *Mtb*RimM_CTD_, their dihedral angles ψ differed by 157.4° (Figure 3D,E). This discrepancy, followed by conformational changes of other downstream residues, directly rendered the β4-β5 loop in *Tth*RimM incapable of forming a dextro-fold and consequently the hydrogen bond. Additionally, taking the conservative V154, V157 and L159 in β5, and V164 and I166 in β6 of *Mtb*RimM_CTD_ as references, the shorter β4-β5 loop in *Tth*RimM forced β5 and even β6 to contract toward the center of CTD (Figure 3F,G). It seemed that the β5 and β6 strands of *Mtb*RimM_CTD_ were more extended than *Tth*RimM, supposedly contributing to the relative higher stability of the β-barrel.

### 3.3. Backbone Relaxation Measurements of MtbRimM_CTD_

**Figure 4:**
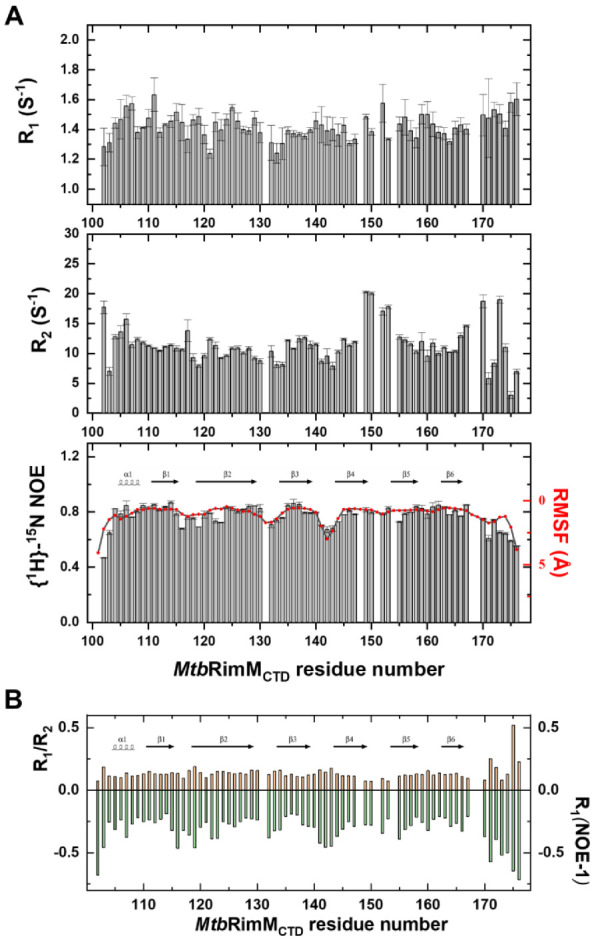
Nuclear magnetic resonance (NMR) relaxation measurements of backbone amide groups in *Mtb*RimM_CTD_. (**A**) Plots of backbone amide dynamics parameters R_1_ (upper panel), R_2_ (middle panel), and {^1^H}-^15^N heteronuclear steady-state nuclear Overhauser effect (hNOE, lower panel) versus residue number. Root-mean-squared fluctuation (RMSF) per residue calculated from molecular dynamics (MD) simulation is plotted over the hNOE graph, as both parameters reveal fast motion features in line with the secondary structure elements shown above the plot. (**B**) Plot of the R_1_/R_2_ ratio and R_1_(NOE-1) value versus residue number. Cross-relaxation rate σ_HN_ is characterized by R_1_(NOE-1) for clearly comparing with the R_1_/R_2_ ratio. Secondary structure elements are shown above the column plot.

To address dynamics features of *Mtb*RimM_CTD_, we performed NMR relaxation measurements of backbone amide groups to obtain longitudinal relaxation rates (R_1_), transverse relaxation rates (R_2_), and {^1^H}-^15^N heteronuclear steady-state nuclear Overhauser effects (hNOE) (Figure 4A). A total of 68 backbone amide resonances were analyzed to characterize internal motions of *Mtb*RimM_CTD_.

R_1_, R_2_, and hNOE are generally used to reflect residue-specific dynamics of the protein. The global average of hNOEs was 0.77, indicating the compactness of the β-barrel. Overall, residues in the loops exhibited smaller R_2_ and hNOE values than those on the β-sheet, implicated in significant conformational flexibility. Plotting R_1_/R_2_ and cross-relaxation rate against sequence number provided a more intuitive understanding of backbone dynamics (Figure 4B), where the strand-interval loops showed faster dynamics than the β-strands. Exceptions were several residues near the β4-β5 loop (residues 149–153) and the C-terminal tail (D102, H170 and L173), as these residues displayed large R_2_ values disproportional to their due flexibilities. Since the apparent R_2_ contains, if any, a conformational exchange term R_ex_ [42], these loop residues were likely to be involved in internal motion on the μs-ms timescale.

The β-sheet included most of the dynamically stable residues according to the relaxation data (Figure 4A,B). As expected, the residues forming the hydrophobic core benefited from the clustering of their non-polar side chains, and showed better rigidity than other residues. Only seven residues in the β-sheet (E119, G122, V123, E134, E144, and T155) which were hydrophilic or located on the protein surface had hNOE values lower than the global average, confirming our speculation.

Then, we evaluated the overall rotational correlation time (τ_c_) of *Mtb*RimM_CTD_ to be 5.87 ± 0.40 ns based on the R_2_/R_1_ data of the residues situated in both the α1 helix and β-sheet [43]. Given the rough linear correlation between τ_c_ and molecular weight (M_w_) on basis of empirical measurements [43], the molecular weight of *Mtb*RimM_CTD_ was estimated to be 9.90 kDa. This estimated M_w_ well conformed to the theoretical M_w_ of recombinant *Mtb*RimM_CTD_ (9.28 kDa), implying that this protein existed in solution as a monomer.

### 3.4. Model-Free Analysis for Backbone Dynamics of MtbRimM_CTD_

**Figure 5:**
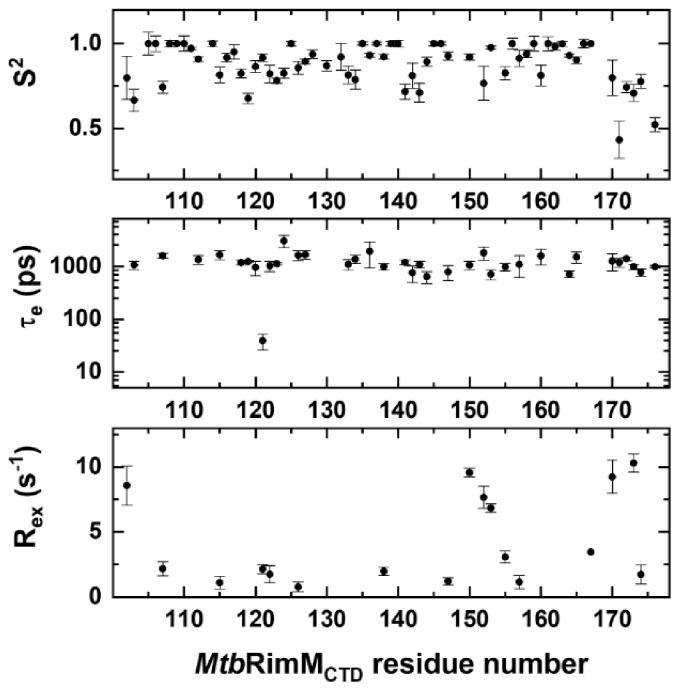
Dynamics parameters of *Mtb*RimM_CTD_ obtained from Model-free analysis of NMR relaxation data. Residue-specific dynamics parameters S^2^ (**upper panel**), τ_e_ (**middle panel**), and R_ex_ (**lower panel**) are plotted per residue. Residues with either lower τ_e_ values or R_ex_ values than their respective errors are not presented in the graphs.

To further comprehend dynamics features of *Mtb*RimM_CTD_, we calculated residue-specific dynamics parameters including the generalized order parameter S^2^, the correlation time of internal motions τ_e_, and the conformational exchange rate R_ex_, based on the measured R_1_, R_2_, and hNOE values by using the FAST-Modelfree program [26,44,45]. We adopted an axially-symmetric rotational diffusion tensor to initiate the iterations. The overall rotational correlation time τ_c_ was fitted to be 6.19 ± 0.03 ns, which was in good agreement with our previous outcome based on the R_2_/R_1_ ratios, with errors taken into account. The critical diffusion tensor parameter D_ratio_ was determined to be 1.05, implying that this protein could be dynamically described using a globular model. A detailed graph for these dynamics parameters is shown in Figure 5. As A131, R151, and three prolines were absent in the 2D NMR spectra for relaxation measurements, 65 out of 71 residues were successfully assigned to different motional models.

The average value of S^2^ was 0.87, indicating that *Mtb*RimM_CTD_ adopted a rigid fold. Collectively, the S^2^ values showed fluctuations consistent with the relaxation data. In particular, the residues in α1 helix except for Q107 exhibited the highest S^2^ value of 1, while Q107 displayed apparent flexibility (S^2^ = 0.743 ± 0.035) (Figure 5). Furthermore, Q107 was the unique residue within α1 helix of which the motion pattern required both τ_e_ and R_ex_ values to describe, suggesting this residue underwent fast internal motion (ps-ns) and intermediate conformation exchange (μs-ms). Moreover, 7 out of 11 residues in β2 strand, the longest β-strand, displayed S^2^ values lower than 0.9 and presented nanosecond-timescale internal motions. Note that each half of the long β2 strand was stabilized by the shorter β1 and β3 strands via hydrogen bonding, respectively (Figure 2A).

In essence, R_ex_ characterizes conformational exchange on the μs-ms timescale if applicable and exists as a linear term of R_2_ [42]. Reevaluation of R_2_ by stripping it of possible R_ex_ was thus feasible for the loop residues with excessive R_2_ rates (D102, H170, and L173 in the terminal loops, and V150, A152, and I153 in the β4-β5 loop). These residues showed high R_ex_ values up to 10.3 s^−1^, signifying that their intrinsic R_2_ values were overestimated (Figure 5). Furthermore, the R_ex_ values in the fragment of V150-V157 exhibited a gradual downward trend, implying that the β4-β5 loop experienced overall conformational exchange, which was primarily stabilized by the downstream β5 strand.

### 3.5. MD Simulations of MtbRimM_CTD_

We further explored the structural stability of *Mtb*RimM_CTD_ based on dynamics enlightenment by the NMR relaxation data. To this end, we performed a 120-ns molecular dynamics (MD) simulation starting with the determined solution structure. Indeed, the root-mean-squared fluctuation (RMSF) of backbone amide atoms (N-H) in residues well matched the experiment-derived hNOEs, especially for the inter-strand loops (Figure 4A). The average value of RMSF reached 1.08 Å, highlighting the rigidity of *Mtb*RimM_CTD_ as a whole. Impressively, residue S142 in the β3-β4 loop showed a large RMSF second only to the two terminal residues D101 and E176, conforming to the low conservation of this loop in both sequence and structure.

**Figure 6:**
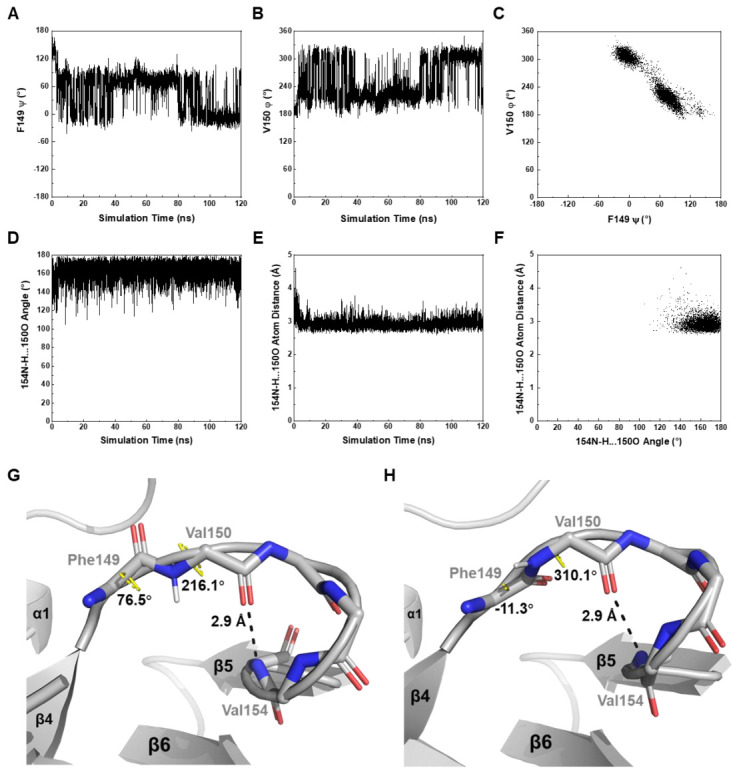
In silico dynamics features of *Mtb*RimM_CTD_ revealed by MD simulation. (**A**,**B**) Fluctuations of critical backbone dihedral angle F149 ψ (**A**) and V150 φ (**B**) in the β4-β5 loop. F149 ψ is scaled to (−180°, 180°) and V150 φ to (0°, 360°) to avoid aliases. (**C**) 2D plot of V150 φ vs. F149 ψ. The connection between F149 and V150 adopts two major orientations, as shown correspondingly in the plot. (**D**–**F**) Stability of the hydrogen bond (V154)N-H…O(V150) indicated in Figure 3D. Both the hydrogen bond angle (**D**) and hydrogen bond length (**E**) remain almost constant in the MD simulation. The 2D plot of hydrogen bond length vs. hydrogen bond angle (**F**) is also implicated in a stable hydrogen bond. (**G**,**H**) Structural snapshots for depicting the motion of the β4-β5 loop at two simulation time of 78.14 ns (**G**) and 106.10 ns (**H**). V150 φ-F149 ψ exhibits two pairs of typical values, while the downstream helix-like fold undergoes motion as an undistorted entity. Hydrogen atoms are hided except for the V150 backbone amide ^1^H atom in each frame.

The above-described model-free analysis revealed that the β4-β5 loop was subjugated to intermediate conformational exchange. During the MD simulation, we observed that the dihedral angles ψ of F149 and φ of V150 both had two different values (Figure 6A–C), while those for the residues 151–155 showed merely minor fluctuations (Figures S2 and S3). Notably, the ψ distinction of F149 influenced the structure of the β4-β5 loop, as indicated by the local structural distinction between *Mtb*RimM_CTD_ and *Tth*RimM CTD. To examine whether the helix-like fold of the β4-β5 loop could undergo loosening, we accessed the time evolution of the V154–V150 hydrogen bond. Intriguingly, the bond length and the N-H…O bond angle remained practically stable throughout the MD simulation (Figure 6D–F), depicting the helix-like structure of the β4-β5 loop as a stable entity. Together, these restrictions described a model in which the conformational exchange of the β4-β5 loop was preferably integral than residue-wise (Figure 6G,H).

The 120-ns MD simulation also provided insight into stabilities of the secondary structure elements contained in *Mtb*RimM_CTD_. As shown in Figure S4, the α-helix and β-strands were generally stable during the simulation. Several inter-strand loops formed turns, further consolidating the rigidity of the protein structure. These MD assessments, combined with the determined structure and NMR relaxation data described above, were indicative of overall well-folded *Mtb*RimM_CTD_ in solution, in contrast to the partly folded *Tth*RimM CTD in solution [13].

### 3.6. Affinity Assessment and Binding Sites Mapping of MtbRimM_CTD_ against S19

**Figure 7:**
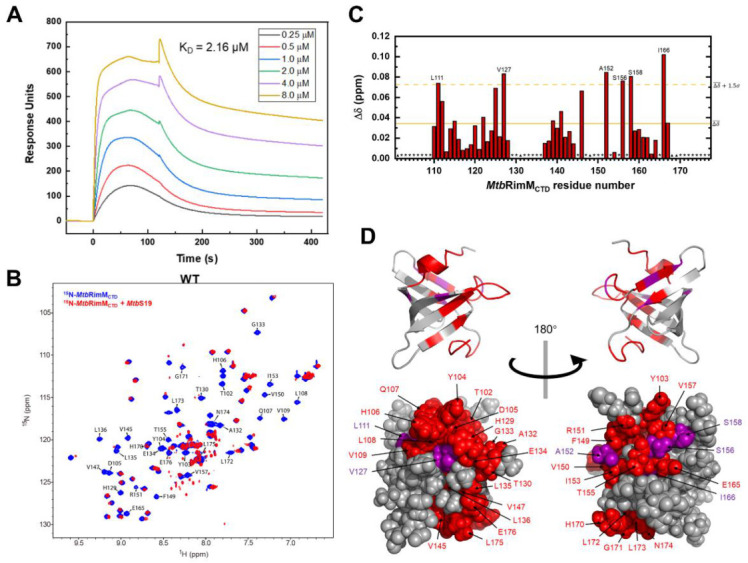
Interaction between *Mtb*RimM_CTD_ and *Mtb*S19. (**A**) Surface plasmon resonance (SPR) affinity assay of *Mtb*RimM_CTD_ binding S19 at serial concentrations. Blank control had been deducted from the serial data. (**B**) Overlapped ^1^H-^15^N heteronuclear singular quantum correlation (HSQC) spectra of ^15^N-labeled *Mtb*RimM_CTD_ alone (blue) and in presence of equimolar *Mtb*S19 (red) for NMR titration assay. Peaks experiencing broadening-induced disappearance are indicated. (**C**) Plot of chemical shift perturbations (CSPs, Δδ) of backbone amide groups. The mean value is indicated by a solid line, and the mean value plus 1.5 standard deviations by a dashed line. Asterisks indicate residues with disappear peaks at the titration destination, while triangles denote residues with invisible resonances before the titration, including D101, A131, and three prolines (residues 148, 168, and 169). (**D**) Mapping the binding surface to the 3D structure of *Mtb*RimM_CTD_. Disappeared peaks are colored in red, and peaks with large CSPs (above the dashed line in (**C**)) in purple. Upper and lower panels are cartoon and sphere depictions of the structure, respectively.

Based on the determined structure of *Mtb*RimM_CTD_, we exploited the interaction between *Mtb*RimM_CTD_ and S19. Thus, we obtained the recombinant S19 protein from *Mtb* (residues 1–93, termed *Mtb*S19), and performed SPR assays to assess the affinity of *Mtb*RimM_CTD_ for binding S19. The dissociation constant (K_D_) of the *Mtb*RimM_CTD_–S19 interaction was measured to be 2.16 μM (Figure 7A), indicative of an intermediate-range interaction. Then, we conducted the equimolar NMR titration of S19 to *Mtb*RimM_CTD_. By comparing the ^1^H-^15^N HSQC spectra of *Mtb*RimM_CTD_ with and without S19, we observed major peak changes (displacement, line broadening, and vanishment) related to backbone amide groups of *Mtb*RimM_CTD_, which indicated an interaction of mM-μM magnitude conforming to the result of SPR assay (Figure 7B). Vanished resonances were associated with the following residues (102–109, 129–136, 145, 147–151, 153, 155, 157, 165, and 170–176), implicated in intermediate conformational exchanges once S19 binding (Figure 7A). We plotted backbone amide chemical shift perturbations (CSPs) of the remaining peaks, and marked the residues with significant CSPs (Figure 7C). Mapping the significantly changed residues to the 3D structure of *Mtb*RimM_CTD_ showed that the S19 binding sites encompassed nearly one-half of the β-barrel (Figure 7D).

### 3.7. Molecular Docking of the MtbRimM_CTD_–S19 Complex

To establish a structural model of the *Mtb*RimM_CTD_–S19 complex, we carried out flexible protein–protein docking of S19 into *Mtb*RimM_CTD_. Although the *Mtb*S19 structure is available as a part of the 70S ribosome structure (PDB: 5V93) [46], residues 84–93 in the C-terminal tail of S19 are absent in the ribosome structure. Given that the corresponding fragment in *Tth*S19 directly contacts *Tth*RimM as displayed in the 3D structure of the *Tth*RimM–S19 complex (PDB: 3A1P), using the structural component of *Mtb*S19 contained in the 5V93 structure as a template might harm the confidence level of the molecular docking. Considering that S19-based structural superposition between the 5V93 and 3A1P structures gave a small backbone RMSD of 1.08 Å (Figure S5), we used the structural component of *Tth*S19 contained in the 3A1P structure as a template to build the structural model of *Mtb*S19. With this structural model and the solution structure of *Mtb*RimM_CTD_, we established the structural model of the *Mtb*RimM_CTD_–S19 complex by molecular docking.

**Figure 8:**
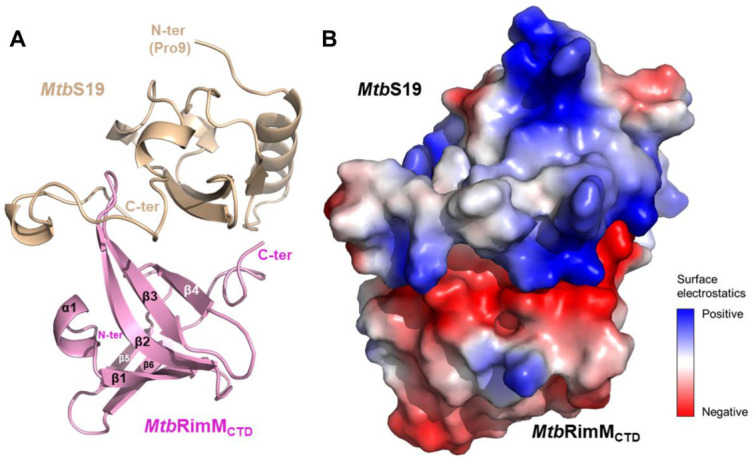
Molecular docking model of the *Mtb*RimM_CTD_–S19 complex. (**A**) Cartoon depiction of the docking model. The structure of *Mtb*S19 (residues 9–93, light brown) was modeled using the crystal structure of *Tth*RimM-complexed *Tth*S19 (PDB: 3A1P) as the template. (**B**) Surface electrostatic potentials of the docking model. The binding interface is mainly composed of charged residues. Positively charged residues are primarily from the long loop located near the C-terminus of *Mtb*S19, and negatively charged residues mostly from *Mtb*RimM_CTD_.

Out of 30,000 structural models generated for the *Mtb*RimM_CTD_–S19 complex, the one with an optimized interface energy score of −61.563 (hereafter referred to as the docking model) was used for the following assessment (Figure 8A). An inspection into the surface electrostatic potentials of *Mtb*RimM_CTD_ and S19 in the docking model indicated extensive electrostatic potential distributions in the binding interfaces on both proteins (Figure 8B). The negatively charged belt in the *Mtb*RimM_CTD_ surface tightly stuck to the positively charged ring of S19, indicating that electrostatic interaction played a predominant role in *Mtb*RimM_CTD_ binding S19. Several residues in *Mtb*RimM_CTD_ (D105, E126, H129, A132, D143, E144, and R151) were involved in direct interactions with S19 (Figure S6), most being charged residues. Hydrophobic interactions also existed between the exposed non-polar parts of both proteins. These results conformed to the binding sites mapped by NMR titration assay described above, which underlined the crucial role of surface electrostatic interactions in minimizing the interface energy of the *Mtb*RimM_CTD_–S19 complex.

**Figure 9:**
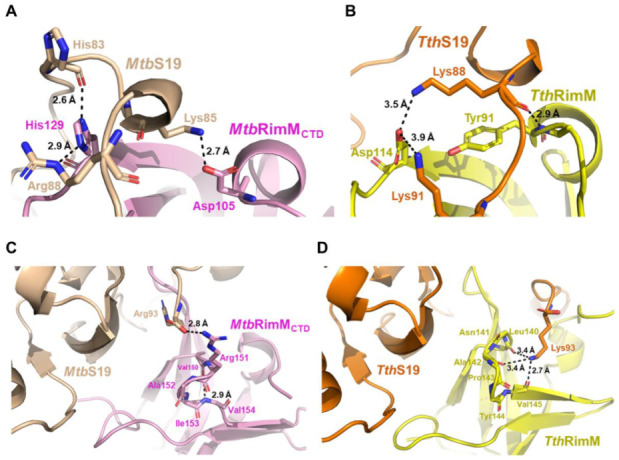
Comparison between the docking model of *Mtb*RimM_CTD_–S19 complex and the crystal structure of *Tth*RimM–S19 complex. (**A**,**B**) Illustration of the stabilized short helix located in the long C-terminal loop of S19 and nearby residues within the docking model of *Mtb*RimM_CTD_–S19 (**A**) or the crystal structure of *Tth*RimM–S19 (PDB: 3A1P) (**B**). Atom distances are shown beside the corresponding black dashed lines. (**C**,**D**) S19 C-terminal residue Arg93 involved in the interaction of *Mtb*S19 with *Mtb*RimM_CTD_ (**C**) or Lys93 in that of *Tth*S19 with *Tth*RimM (**D**). Atom distances are shown beside the corresponding black dashed lines.

Notably, the imidazole group of H129 at the end of the β2 strand in *Mtb*RimM_CTD_ formed hydrogen bonds with both the backbone oxygen of H83 and side-chain amide of R88 in *Mtb*S19, making H129 a critical dual-binder for *Mtb*RimM_CTD_ binding S19 (Figure 9A; Figure S6). Meanwhile, *Mtb*RimM_CTD_ D105 in α1 helix formed a hydrogen bond with *Mtb*S19 K85 in the short helix. Structural superposition displayed that the C-terminal tail of *Mtb*S19 in the docking model of the *Mtb*RimM–S19 complex underwent a displacement relative to the *Tth*RimM–S19 complex. On the other hand, *Tth*RimM D114 (corresponding to *Mtb*RimM_CTD_ H129) also served as a dual-binder, forming two salt bridges with the side chains of *Tth*S19 K88 and K91 (Figure 9B). Furthermore, the carbonyl group of *Tth*RimM Y91 (corresponding to *Mtb*RimM_CTD_ D105) formed a hydrogen bond with the backbone amide group of *Tth*S19 K88. Sequence alignment showed that neither D105 nor H129 in *Mtb*RimM were conserved in other RimM orthologs (Figure 1B). It seemed that *Mtb*RimM D105 and H129 might contribute to the unique pattern of *Mtb*RimM_CTD_ binding S19, adopting different hydrogen binding modes to bind S19 from *Tth*RimM Y91 and D114.

Another distinction between the docking model of *Mtb*RimM_CTD_–S19 and crystal structure of *Tth*RimM–S19 lay in the β4-β5 loop. The oxygen atoms of backbone carbonyls of *Tth*RimM L140, A142, and V145 in the β4-β5 loop encircled the side chain amide atoms of *Tth*S19 K93, upholding the flexible C-terminus (Figure 9D). However, this interaction pattern did not work for *Mtb*RimM_CTD,_ potentially owing to the structural distinction in the β4-β5 loop between *Mtb*RimM_CTD_ and *Tth*RimM. The docking model of *Mtb*RimM–S19 displayed an alternative binding pattern in which a hydrogen bond was formed between the guanidine group of *Mtb*RimM R151 and carboxyl group of *Mtb*S19 R93 (Figure 9C; Figure S6). The structural distinction of the β4-β5 loop, together with the critical charge-reversive substitution at residue 129 (positively charged *Mtb*RimM_CTD_ H129 vs. negatively charged *Tth*RimM D104), might contribute the change in the interaction pattern of the C-terminal tail in *Mtb*S19. In summary, the docking model of *Mtb*RimM_CTD_–S19 was distinguished from the crystal structure of *Tth*RimM–S19 primarily due to structure rearrangements between the two orthologs which were induced by pivotal residue replacements.

### 3.8. Affinity Assessments for MtbRimM_CTD_ Mutants Binding S19

**Figure 10:**
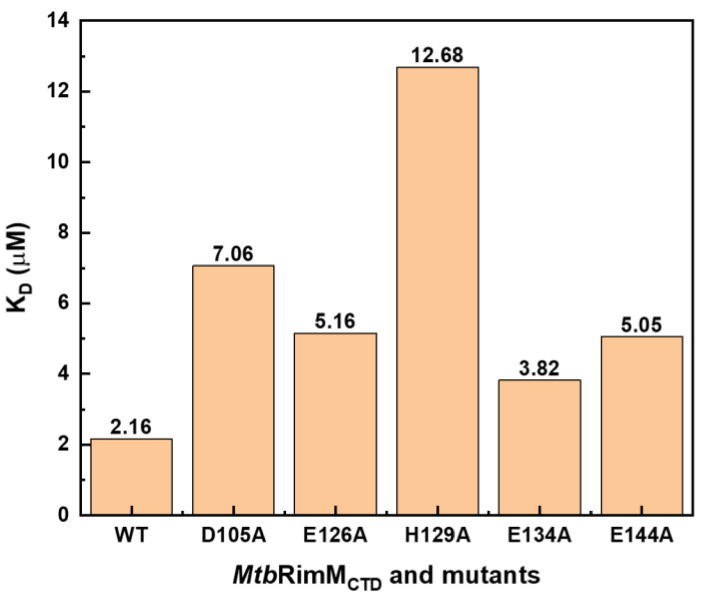
Dissociation constants (K_D_) of wild-type (WT) *Mtb*RimM_CTD_ and its mutants for binding *Mtb*S19 determined by SPR affinity assays.

To verify the reliability of the docking model of *Mtb*RimM_CTD_–S19, we obtained site-directed mutants of *Mtb*RimM_CTD_ at key loci and then measured their affinities for binding *Mtb*S19 by SPR assays in comparison to the wild-type (WT) *Mtb*RimM_CTD_ (Figure S7A–E). Mutation candidates included the nonconserved D105 and H129 considering their potential key roles in the unique interaction pattern of *Mtb*RimM_CTD_ with S19, and the conservative E126, E134, and E144 joining in electrostatic interactions with S19. Since they functioned primarily with their charged side chains according to the docking model of *Mtb*RimM_CTD_–S19, these five residues were subjugated to alanine substitutions accordingly. As a result, all these mutants displayed 0.8–4.9 times increases in their K_D_ values compared to WT, signifying significantly decreased affinities (Figure 10). In particular, H129A exhibited the most considerable change of affinity among the mutants as indicated by the K_D_ values (12.68 μM for H129A vs. 2.16 μM for WT), agreeing with the dual-binder role of *Mtb*RimM H129 in the docking model, despite this nonconserved residue with diverse charges among the orthologs. The second-largest decrease in affinity was observed for *Mtb*RimM D105A binding S19 (K_D_ = 7.06 μM), followed by E144A and E126A. As a highly conservative component of the positively charged belt on the *Mtb*RimM_CTD_ surface but not present on the S19 binding interface of the docking model, the E134A mutant showed the smallest change in K_D_, as expected. These results indicated that the two unique residues D105 and H129 played vital roles in the interaction of *Mtb*RimM_CTD_ with S19, consolidating the creditability of the docking model of *Mtb*RimM_CTD_–S19.

## 4. Discussion

TB is threatening the world healthcare with its MDR variants. Novel drug targets are urgently needed for effective prevention and treatment of MDR-TB. As is known, ribosomes are the organelle responsible for controlling protein biosynthesis. Critical protein components in bacterial ribosomes could be explored as potential drug targets [47]. As one of the regulation factors vital for 30S ribosomal subunit assembly in vivo with S19-binding activities in vitro, RimM could be a potential target for the development of novel drugs against MDR-TB [5,8]. However, the 3D structure of *Mtb*RimM has not yet been determined. Considering that the CTD is primarily responsible for the interaction of *Mtb*RimM with S19, we herein clarified the structural basis of *Mtb*RimM_CTD_ binding S19 by applying several biophysical techniques. Based on the mapped S19 binding sites on *Mtb*RimM_CTD_, we established the docking model of the *Mtb*RimM_CTD_–S19 complex, and identified critical residues significantly contributing to the unique pattern of *Mtb*RimM_CTD_ binding S19.

### 4.1. MtbRimM CTD Is Structurally Independent of NTD and Primarily Responsible for Binding S19

Prior to structure determination, we firstly confirmed structural independence between the CTD and NTD of *Mtb*RimM. A previous study declared the structural independence of *Tth*RimM CTD on NTD by comparing the NMR structure ensemble and protein dynamics of a standalone NTD truncation with the NTD part of the full-length *Tht*RimM [13]. To examine the structural independence, we separately recorded ^1^H-^15^N HSQC spectra on full-length *Mtb*RimM, *Mtb*RimM_NTD_ and *Mtb*RimM_CTD_. Most peaks of both *Mtb*RimM_NTD_ and *Mtb*RimM_CTD_ overlapped well to those belonging to the full-length *Mtb*RimM, implying that the domain truncation almost did not affect the structure of either CTD or NTD (Figure S8). Peaks of the N-terminal helix (residues 102–108) and inter-β-strand loops (F149, V150, L159, and D160) in *Mtb*RimM_CTD_ showed slight shifts relative to full-length *Mtb*RimM. Furthermore, the NMR titration of *Mtb*RimM_NTD_ into *Mtb*RimM_CTD_ and its reverse counterpart did not display observable peak changes (Figure S9). We thereby proved the structural independence between the CTD and NTD of *Mtb*RimM. Additionally, the NMR titration of *Mtb*S19 into *Mtb*RimM_NTD_ did not show observable peak changes (Figure S10), implying that S19 basically did not bind to *Mtb*RimM_NTD_. Together, these results allow us to exploit the structural basis of *Mtb*RimM_CTD_ binding S19 without NTD interference.

### 4.2. Unique Features of β3-β4 and β4-β5 Loops Characterize a Well-Folded MtbRimM_CTD_

The solution structure of *Mtb*RimM_CTD_ is comprised of a six-strand β-barrel and a short α-helix near the N-terminus, characterized by a hydrophobic core and a predominantly negatively charged surface. The tightly packed core consists of conservative non-polar residues mostly located on the six β-strands. The overall rigidity *of Mtb*RimM_CTD_ is reflected by the large all-residue mean S^2^ value of 0.87 (the generalized order parameter), and the small RMSF of 1.08 Å in the MD simulation. Different from well-folded *Mtb*RimM_CTD_, *Tth*RimM CTD is only partly folded in solution, as residues 125–143 showed few medium- or long-range NOEs [13]. However, crystal structures of both free *Tth*RimM (PDB: 2DYI) and S19-complexed full-length *Tth*RimM (PDB: 3A1P) display a CTD of β-barrel fold resembling *Mtb*RimM_CTD_, suggesting that the unfolded part of *Tth*RimM CTD in solution was not stabilized until trapped in a local energy minimum during crystallization. In fact, all five crystal structures of RimM orthologs available in PDB exhibit general structural similarity to *Mtb*RimM_CTD_ despite low sequence identity. Interestingly, the fragment in *Mtb*RimM_CTD_ corresponding to residues 125–143 in *Tth*RimM starts from the end of β3 and ends at the middle of β5, covering the two major conformational discrepancies between the two orthologs in the β3-β4 loop and β4-β5 loop. Moreover, the highly flexible β3-β4 loop of *Tth*RimM CTD showed R_1_ values up to 4 s^−1^ and low hNOE values around 0.5 [13], in sharp contrast to the results obtained from the NMR relaxation measurement of *Mtb*RimM_CTD_. Considering these results and the unique structural features of these two loops described above, the far shorter β3-β4 loop and the β4-β5 loop stabilized by a hydrogen bond (V154–V150) might facilitate the stable folding of *Mtb*RimM_CTD_.

### 4.3. Both β4-β5 Loop and Nonconserved Key Residues Contribute to the Unique Pattern of MtbRimM_CTD_ Binding S19

The docking model of *Mtb*RimM_CTD_–S19 shows an interface containing surface charged residues, where the positively charged C-terminal tail of *Mtb*S19 binds to the negatively charged ring of *Mtb*RimM_CTD_ (Figure 8B). The docking model displays a binding pattern different from that identified from the crystal structure of *Tth*RimM–S19 (PDB: 3A1P). The β4-β5 loop exhibits a smaller possibility to accommodate the C-terminus of *Mtb*S19 via multiple hydrogen bonds, forcing the terminal arginine to relocate to a different side of the 3A1P structure. Notably, conformational exchanges in this loop, which was intrinsically caused by the dihedral angle fluctuations of F149-V150, were revealed by NMR relaxation analysis and also confirmed by MD simulation (Figure 5 and Figure 6A–C). Comparatively, the experiment-based difference of the corresponding dihedral angle between *Mtb*RimM_CTD_ F149 ψ and *Tth*RimM L140 ψ was 157.4°, while the simulation-based fluctuation between the two dihedral angles individually related to two major orientations of *Mtb*RimM_CTD_ F149 ψ or V150 φ was nearly 90° (Figure 6A,B). The orientation distinction, together with the stable hydrogen bond of V154-V150 (Figure 3D), might provide a mechanistic understanding of the unique role of the helix-like β4-β5 loop in *Mtb*RimM_CTD_ binding S19, which is distinct from that in *Tth*RimM binding S19. Additionally, two nonconserved residues D105 and H129 in *Mtb*RimM_CTD_ could cause alteration of binding destination on the C-terminal tail of S19 (Figure 8A,B). Although *Mtb*RimM_CTD_ H129 displays reverse charge relative to its counterparts in RimM orthologs (e.g., D114 in *Tth*RimM), its dual-binder function works well via the hydrogen bond rather than the salt bridge adopted by *Tth*RimM D114, as evidenced by the largest affinity decrease of the H129A mutant observed by SPR assays. Hence, the docking model reveals a unique pattern of *Mtb*RimM_CTD_ binding S19.

Notably, some residues in *Mtb*RimM_CTD_ that displayed peak broadening in the NMR titration experiment seemed not to directly contact *Mtb*S19 in the docking model of the *Mtb*RimM_CTD_–S19 complex. One of the potential reasons is that not only *Mtb*RimM_CTD_–S19 interaction directly cause significant line broadening in residues on the S19-binding surface of *Mtb*RimM_CTD_, but also conformational changes induced by either S19 binding or non-specific interactions could cause observable line broadening in several residues around the S19-binding surface. Expectedly, single-site mutation experiments on a case-by-case basis can help distinguishing the non-specific interactions from the specific interactions. Furthermore, a previous work provides comprehensive methods for identifying nonspecific interactions in the protein-ligand complex, especially the ligand-induced modulation [48].

### 4.4. Partial Affinity Decrease of MtbRimM_CTD_–S19 by Single-Site Mutation Calls for Efficient Binding Inhibitors

While we have characterized the structural and interaction-related properties of *Mtb*RimM_CTD_, some intriguing facts might hamper efforts of *Mtb*RimM-based drug design. As described above, the single-site mutations of five residues (D105A, E126A, H129A, E134A, and E144A), no matter they were conservative residues or not, observably reduced but did not neutralize the affinity of *Mtb*RimM_CTD_ binding S19. This result suggests that single-site mutation is insufficient for full inhibition of the *Mtb*RimM_CTD_–S19 interaction. The logic behind this observation might lie in fault tolerance of RimM introduced during species evolution. Interestingly, even though RimM CTDs exhibit low conservation except for the residues forming the hydrophobic core, S19 orthologs among several bacterial species are highly conservative (Figure S11). In comparison, the human 40S ribosomal protein S15 shares a lower sequence identity with *Mtb*S19 despite both proteins belong to the S19 protein family. Thus, relative low side effects on human hosts could be expected for potential drugs designed to specifically target *Mycobacterium tuberculosis* and significantly breaking the *Mtb*RimM–S19 interaction. Additionally, the design of new anti-TB drugs eliminating the *Mtb*RimM_CTD_–S19 interaction should focus on aiming key residues on the S19-binding pockets in *Mtb*RimM_CTD_, or alternatively, on targeting the more conservative *Mtb*S19.

Furthermore, we predicted the preliminary druggability of the *Mtb*RimM_CTD_–S19 complex by using the PockDrug webserver [49] based on the docking model of *Mtb*RimM_CTD_–S19. The prediction identified two potential pockets, showing moderate druggability probabilities for both pockets (Figure S12, Table S1). Notably, pocket 1 involves *Mtb*RimM_CTD_ E126 and H129 and *Mtb*S19 H83, and pocket 2 involves *Mtb*RimM_CTD_ R151 and *Mtb*S19 R93. These residues highly cover with the key residues on the interaction interface between *Mtb*RimM_CTD_ and S19, implicated in the pharmacal significance of *Mtb*RimM as a potential anti-TB drug target.

Expectedly, lead compounds capable of profoundly interfering with the hydrogen bonds and/or electrostatic interactions might inhibit the *Mtb*RimM–S19 interaction and break this complex. Moreover, the convex hull volumes of both predicted drug pockets are in the order of 100 Å^3^ (Table S1), indicative of the accommodation ability of small ligands. Taken together, these considerations imply that hydrophilic, charged, and slim molecules with capacities of blocking both drug pockets could be explored to be potential drugs targeting the *Mtb*RimM–S19 interaction.

As the heteronuclear 3D NMR spectra recorded on full-length *Mtb*RimM exhibited severe peak overlap or peak broaden even disappearance, it is a difficult task to complete resonance assignments and structural determination for the full-length protein in solution. Nevertheless, we have demonstrated the structural independence between the NTD and CTD of *Mtb*RimM. The current study on the standalone *Mtb*RimM_CTD_ will facilitate the future study on solution structure, dynamics and intermolecular interaction of full-length *Mtb*RimM. Our results provide new insights into the molecular mechanisms of the RimM function regarding the assembly of S19 into the ribosome. Moreover, the structural basis of *Mtb*RimM_CTD_ binding S19 revealed in this study may be beneficial to the development of novel drugs against MDR-TB.

## Figures and Tables

**Table 1:** NMR restraints and structural statistics for *Mtb*RimM_CTD_.

NMR Distance and Dihedral Angle Constraints		Values
Total ambiguous distance restraints		117
Unambiguous distance restraints	Intra-residual	413
	Sequential (|i − j| = 1)	226
	Short range (2 ≤ |i − j| ≤ 3)	80
	Medium range (4 ≤ |i − j| ≤ 5)	50
	Long range (|i − j| > 5)	200
	Total	969
Dihedral angle restraints	φ	63
	ψ	63
	Total	126
**Structural Statistics**		
Mean restraint violations	Distance restraint violations (>0.3 Å)	0
	Dihedral restraint violations (>5°)	0
Average root-mean-squared-deviation (RMSD) (Å) to mean structure (residues 103–173)	Backbone RMSD	0.23 ± 0.05
	Heavy atoms RMSD	0.85 ± 0.10
Ramachandran plot statistics ^1^	Residues in favored regions	85.0% ± 2.0%
	Residues in allowed regions	14.0% ± 2.0%
	Residues in disallowed regions	1.0% ± 1.0%

^1^ Accessed from PDB structure validation report.

## Data Availability

The atomic coordinate file of *Mtb*RimM_CTD_ presented in this study is openly available in the Protein Data Bank Japan (https://pdbj.org/) at https://doi.org/10.2210/pdb7CQ1/pdb, reference number 7CQ1.

## Supplementary Materials

The following are available online at https://www.mdpi.com/article/10.3390/biom11040597/s1, Figure S1: Structural superpositions between *Mtb*RimM_CTD_ and RimM orthologs with known structures, Figure S2: Time evolutions of the backbone dihedral angle φ of residues 151–155 in *Mtb*RimM_CTD_, Figure S3: Time evolutions of the backbone dihedral angle ψ of residues 150–155 in *Mtb*RimM_CTD_, Figure S4: Percentage of secondary structural propensities per residue predicted from the MD simulation, Figure S5: Structural superposition between the *Tth*RimM-complexed *Tth*S19 (PDB: 3A1P) and *Mtb*S19 incorporated into the capreomycin-bound 70S ribosome from *M. tuberculosis* (PDB: 5V93), Figure S6: Illustration of intermolecular interactions identified from the docking model of the *Mtb*RimM_CTD_–S19 complex, Figure S7: SPR affinity assays of *Mtb*RimM_CTD_ mutants binding *Mtb*S19 at serial concentrations, Figure S8: Overlapped ^1^H-^15^N HSQC spectra of *Mtb*RimM (black), *Mtb*RimM_NTD_ (blue), and *Mtb*RimM_CTD_ (red), Figure S9: Mutual NMR titration assays between *Mtb*RimM_CTD_ and *Mtb*RimM_NTD_, Figure S10: NMR titration assay of *Mtb*RimM_NTD_ binding S19, Figure S11: Sequence alignments among *Mtb*S19 and S19 orthologs corresponding to those for RimM CTDs, Figure S12: Prediction of potential drug pockets in the *Mtb*RimM_CTD_–S19 docking model via PockDrug server, Table S1: Parameters of predicted drug pockets in the *Mtb*RimM_CTD_–S19 docking model via PockDrug server.
