# Supplementary material for: Structural Basis for the C-Terminal Domain of Mycobacterium tuberculosis Ribosome Maturation Factor RimM to Bind Ribosomal Protein S19
Source: Biomolecules. 2021 Apr 18;11(4):597. doi: 10.3390/biom11040597 (PMC8073977; doi:10.3390/biom11040597)
Supplement: Supplementary file 1 [file biomolecules-11-00597-s001.zip › biomolecules-1184587-supplementary.pdf]

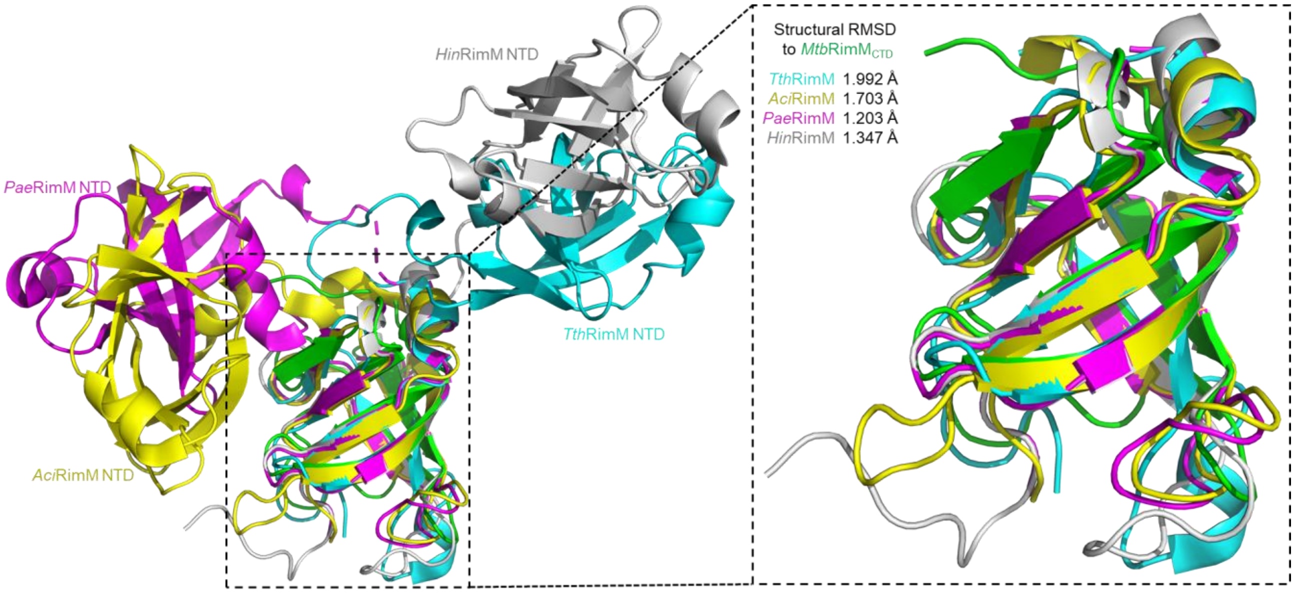

Figure S1. Structural superpositions between *Mtb*RimM$_{CTD}$ and RimM orthologs with known structures. Crystal structures of *Tth*RimM (cyan, PDB: 2DYI), *Aci*RimM (yellow, PDB: 2QGG), *Pae*RimM (purple, PDB: 2F1L), and *Hin*RimM (gray, PDB: 3H9N) were superposed to *Mtb*RimM$_{CTD}$ (green) by C$_{\alpha}$ atoms using Pymol. Left panel, the structures of *Mtb*RimM$_{CTD}$ and the full-length orthologs after superpositions; right panel, magnified structures of *Mtb*RimM$_{CTD}$ and CTDs of RimM orthologs, with their corresponding coordinate RMSDs to *Mtb*RimM$_{CTD}$.

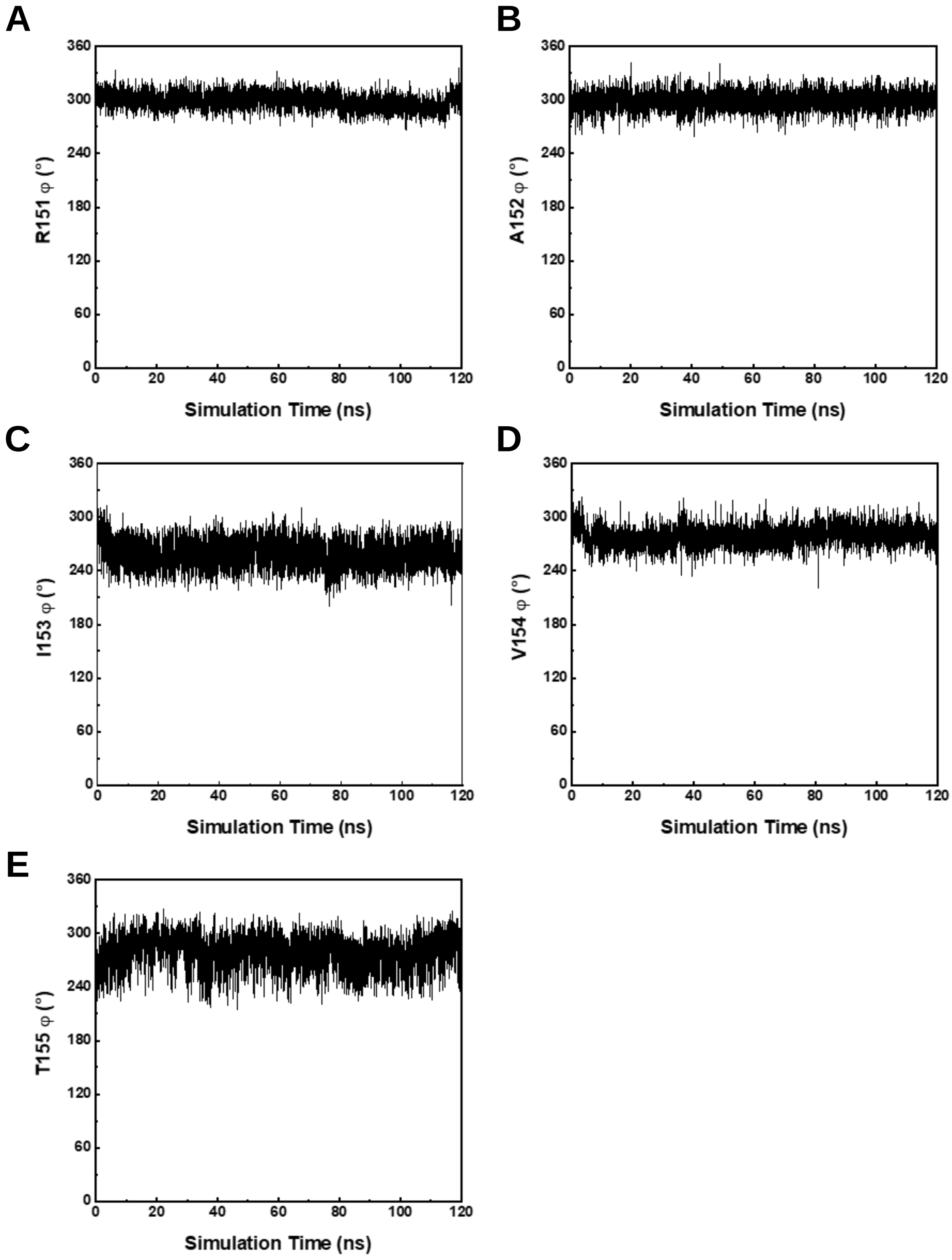

Figure S2. Time evolutions of the backbone dihedral angle φ of residues 151-155 in *Mtb*RimM$_{\mathbf{CTD}}$.

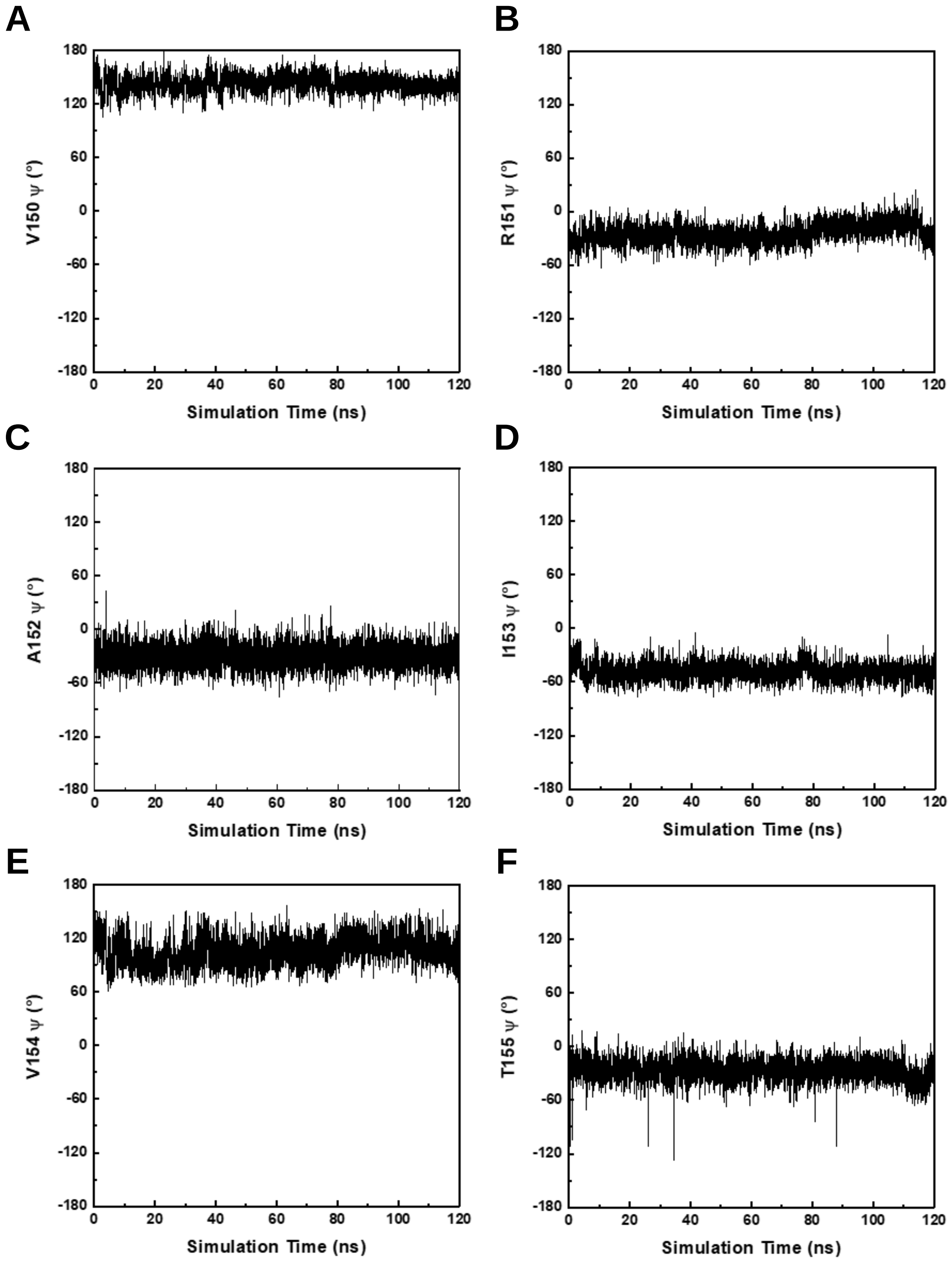

Figure S3. Time evolutions of the backbone dihedral angle ψ of residues 150-155 in *Mtb*RimM$_{CTD}$.

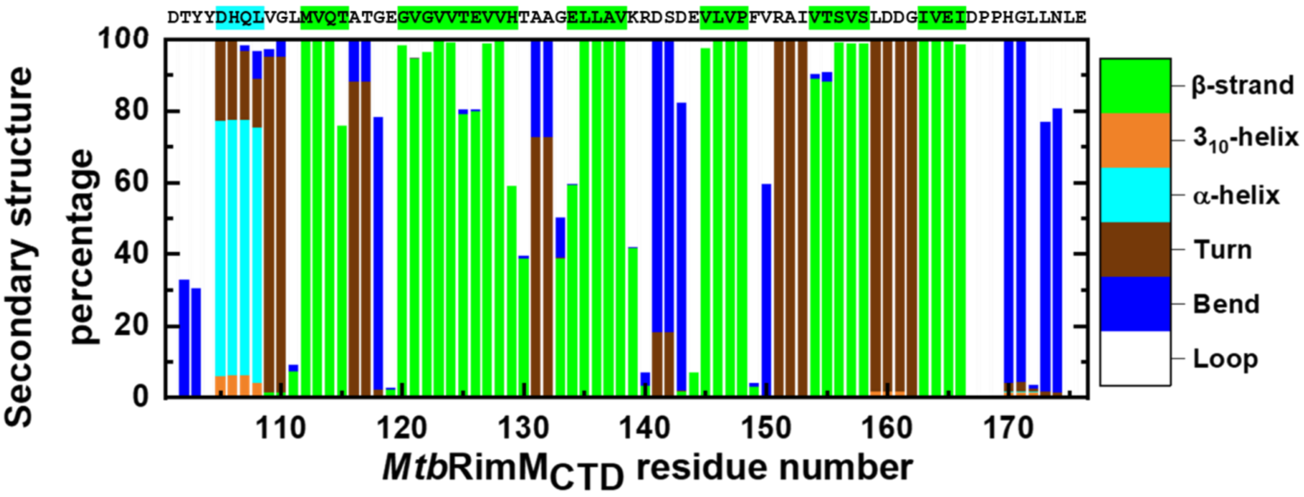

Figure S4. Percentage of secondary structural propensities per residue predicted from the MD simulation. The secondary structure elements (α-helices and β-strands) are highlighted on the protein sequence with the same color pattern displayed in the main plot.

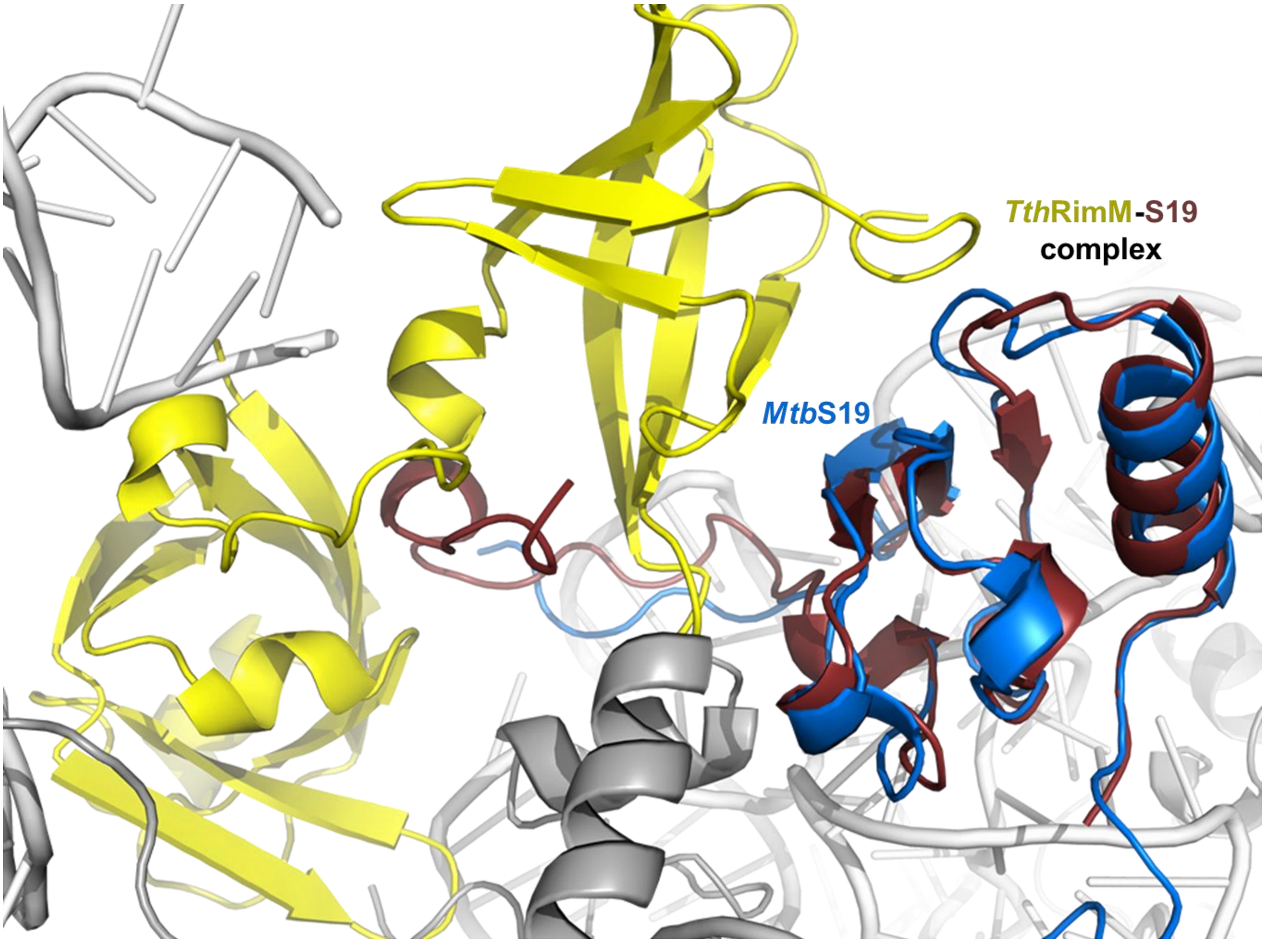

Figure S5. Structural superposition between the *Tth*RimM-complexed *Tth*S19 (PDB: 3A1P) and *Mtb*S19 incorporated into the capreomycin-bound 70S ribosome from *M. tuberculosis* (PDB: 5V93). The structure of *Tth*S19 (ruby) complexed with *Tth*RimM (yellow) showed an RMSD of 1.08 Å to that of *Mtb*S19 (marine blue) in the ribosome, indicating structural conservation among S19 orthologs. Other nucleic acids or proteins in the ribosome are colored in white or gray.

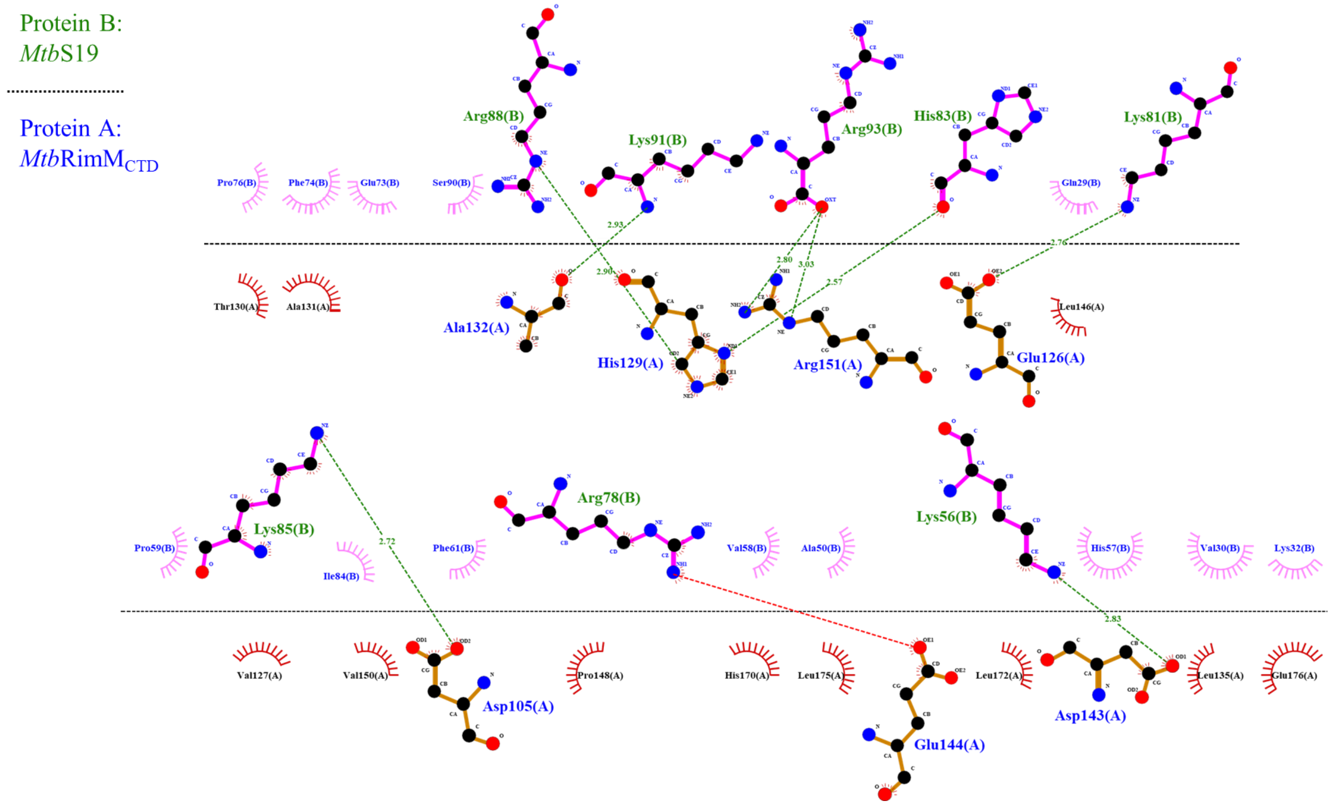

Figure S6. Illustration of intermolecular interactions identified from the docking model of the *Mtb*RimM$_{CTD}$-S19 complex.

This graph was generated by the LigPlot+ program. Residues beneath and above each horizonal dividing line are from *Mtb*RimM$_{CTD}$ (indicated as Protein A) and *Mtb*S19 (indicated as Protein B), respectively. Green and red dashed lines denote hydrogen bonds and salt bridges, respectively. Residues or atoms involved in hydrophobic interactions are circled by an arc decorated with sticks.

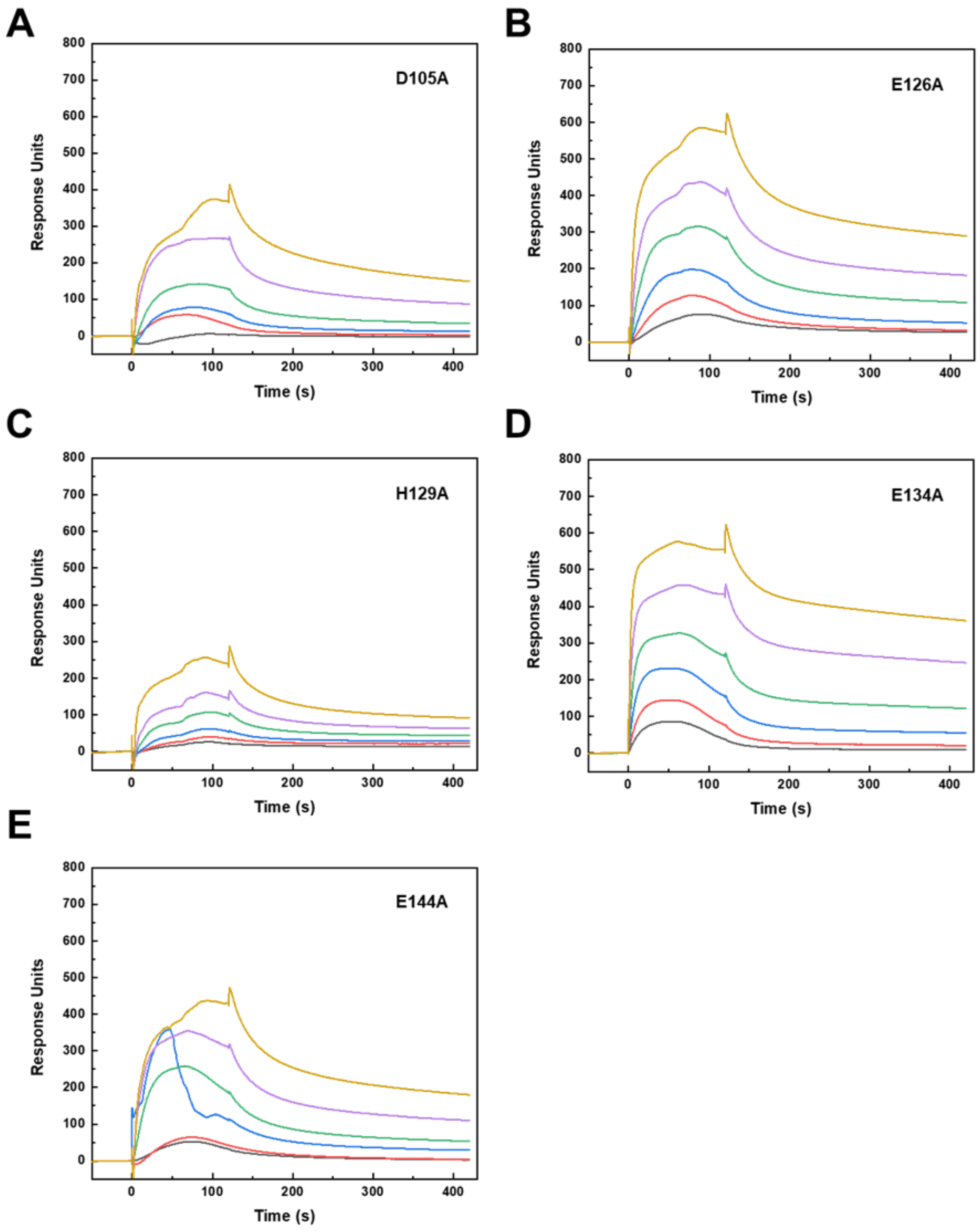

Figure S7. SPR affinity assays of *Mtb*RimM$_{CTD}$ mutants binding *Mtb*S19 at serial concentrations.

(A-E) SPR assay curves of D105A (A), E126A (B), H129A (C), E134A (D) and E144A (E). The following concentrations (μM) were used for *Mtb*S19: 0.25 (black curve), 0.5 (red), 1.0 (blue), 2.0 (green), 4.0 (purple), and 8.0 (orange). Blank control had been deducted.

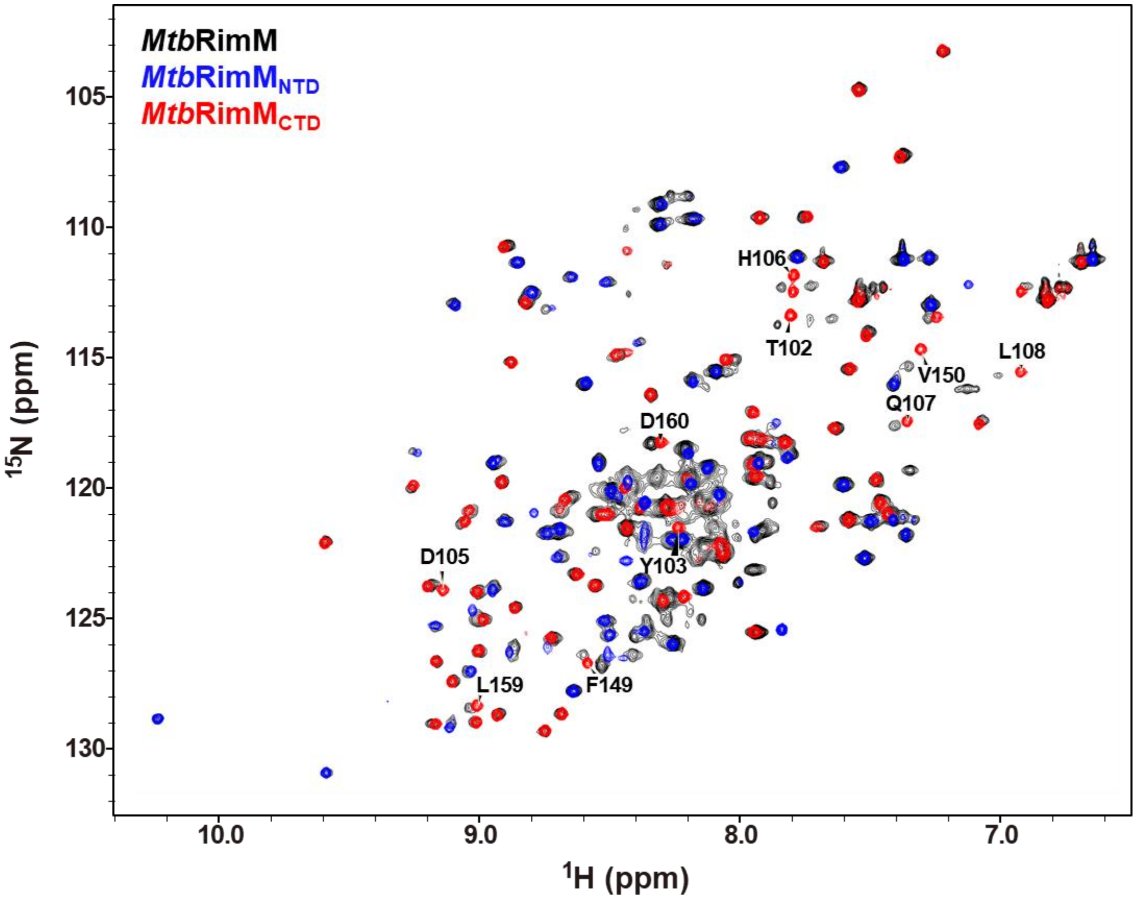

Figure S8. Overlapped ^{1}H-^{15}N HSQC spectra of *Mtb*RimM (black), *Mtb*RimM$_{NTD}$ (blue) and *Mtb*RimM$_{CTD}$ (red).

Residues of *Mtb*RimM$_{CTD}$ with observable peak shifts relative to *Mtb*RimM are labeled in the spectra.

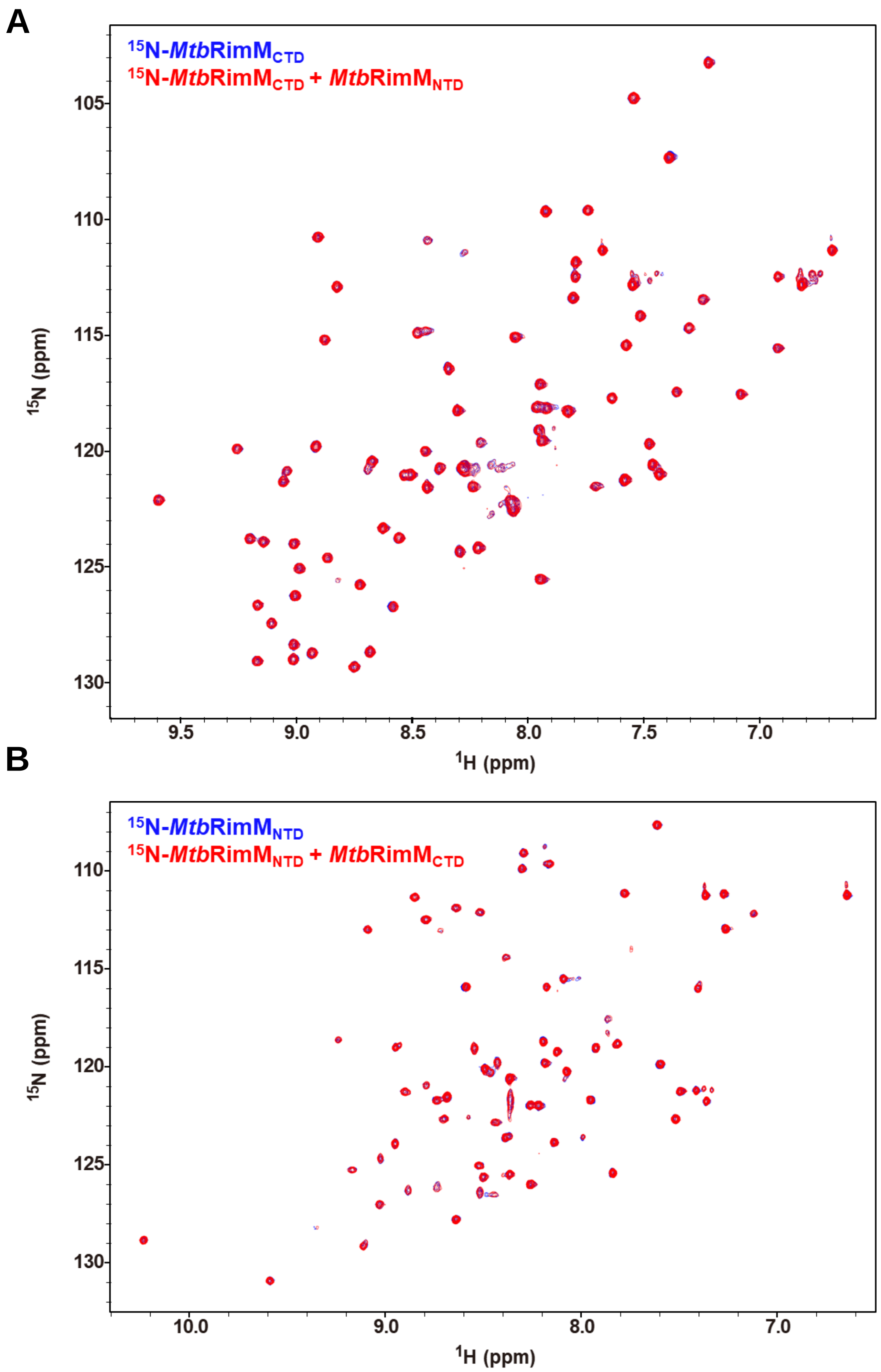

Figure S9. Mutual NMR titration assays between *Mtb*RimM$_{CTD}$ and *Mtb*RimM$_{NTD}$.

(A) Overlapped ^{1}H-^{15}N HSQC spectra of ^{15}N-labeled *Mtb*RimM$_{CTD}$ alone (blue) and in presence of equimolar *Mtb*RimM$_{NTD}$ (red).

(B) Overlapped ^{1}H-^{15}N HSQC spectra of ^{15}N-labeled *Mtb*RimM$_{NTD}$ alone (blue) and in presence of equimolar *Mtb*RimM$_{CTD}$ (red).

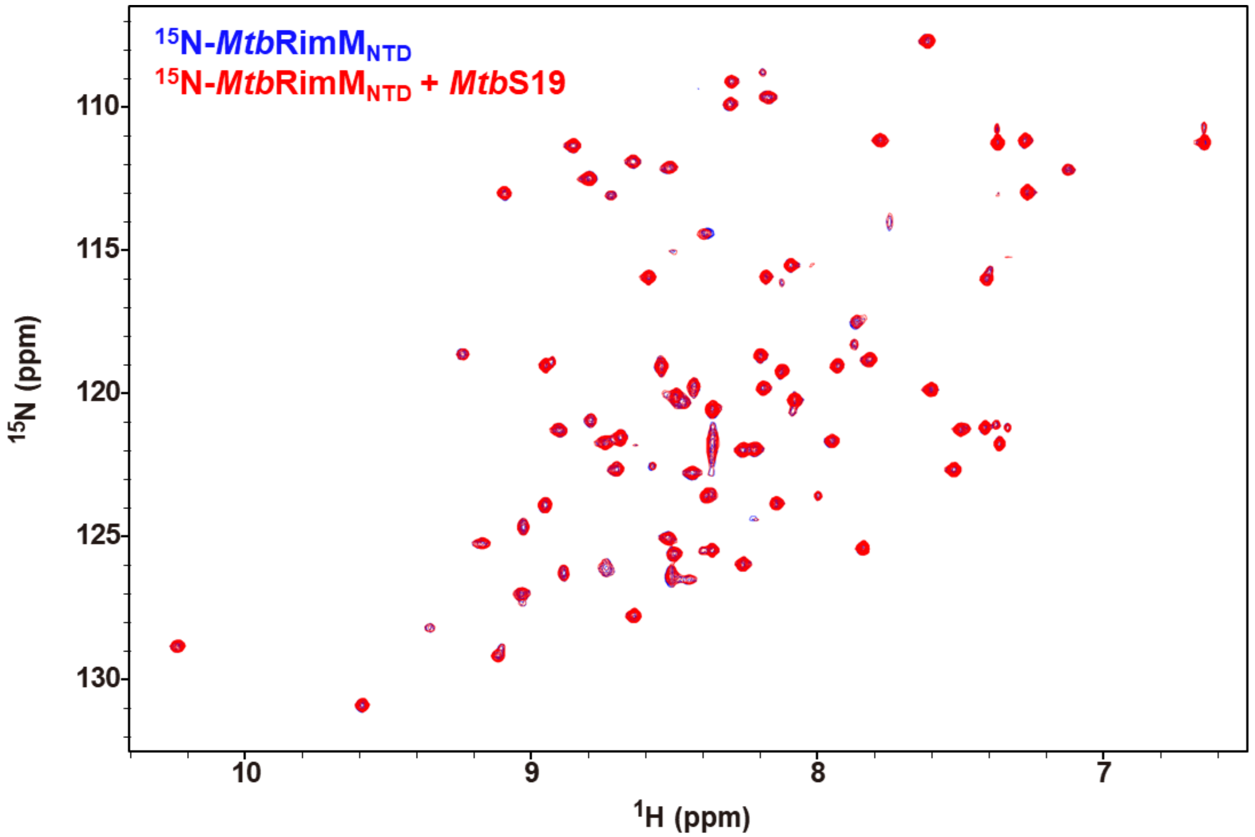

Figure S10. NMR titration assay of *Mtb*RimM$_{NTD}$ binding S19.

This plot displays overlapped ^{1}H-^{15}N HSQC spectra of ^{15}N-labeled *Mtb*RimM$_{NTD}$ alone (blue) and in presence of equimolar *Mtb*S19 (red).

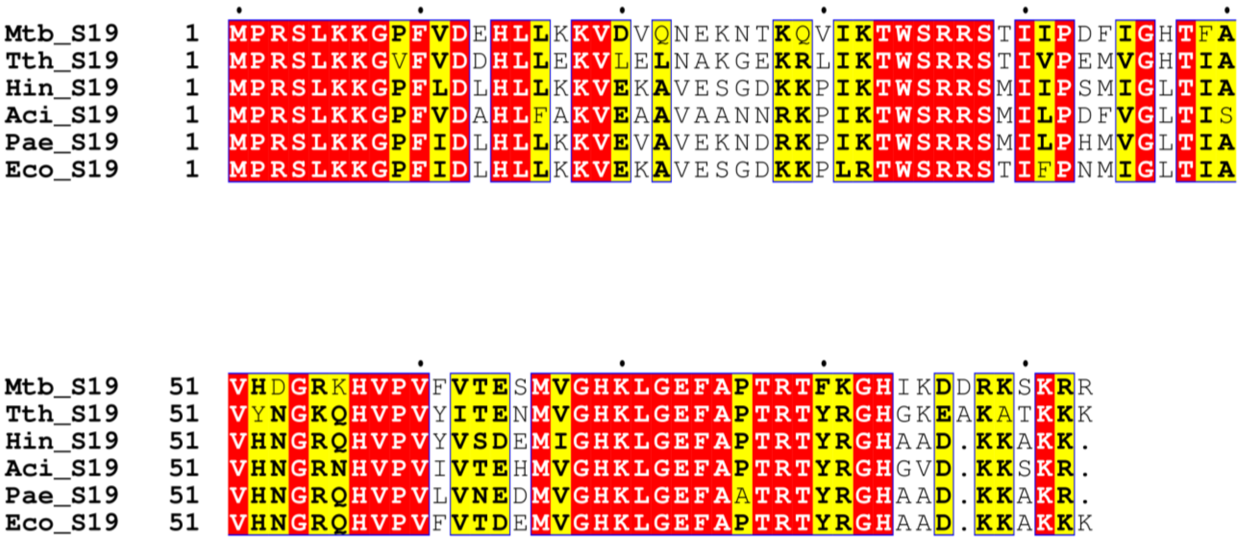

Figure S11. Sequence alignments among *Mtb*S19 and S19 orthologs corresponding to those for RimM CTDs.

This plot was generated by using Clustal X2 and Espript 3.0, where identical residues are colored in red, and similar residues in yellow. Numerical indicators for the first residue and every ten residue (10, 20, 30, …, 90) are shown as black dots above the alignments for *Mtb*S19.

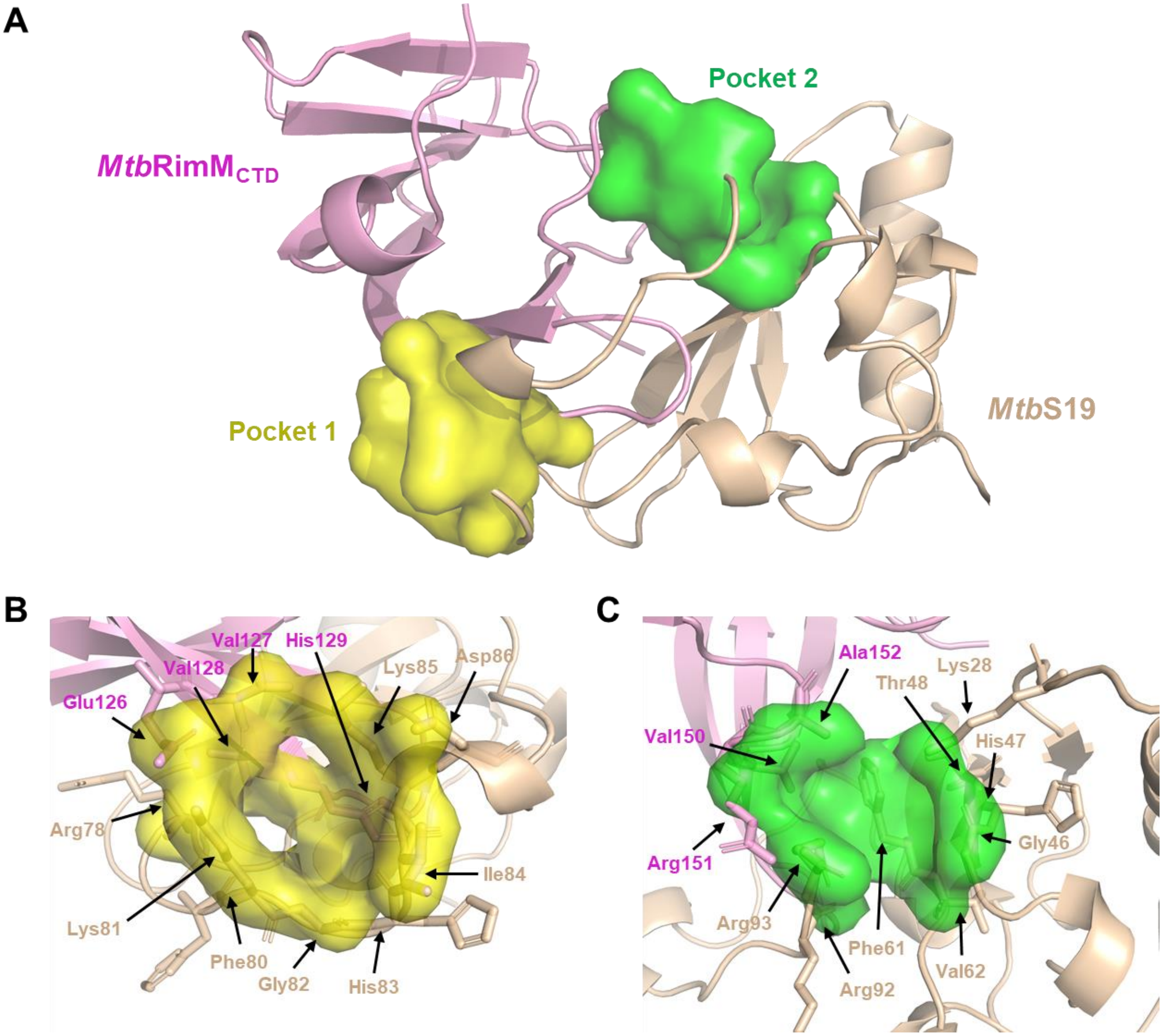

Figure S12. Prediction of potential drug pockets in the *Mtb*RimM$_{CTD}$-S19 docking model via PockDrug server.

(A) The global positions of the two predicted drug pockets. *Mtb*RimM$_{CTD}$ and its residues are hereafter indicated in pink, and *Mtb*S19 and its residues in light brown.

(B) Surface depiction of Pocket 1. Note that critical binding sites *Mtb*RimM$_{CTD}$ E126 and H129 and *Mtb*S19 H83 are involved.

(C) Surface depiction of Pocket 2. Note that critical binding sites *Mtb*RimM$_{CTD}$ R151 and *Mtb*S19 R93 are involved.

Table S1. Parameters of predicted drug pockets in the *Mtb*RimM$_{CTD}$-S19 docking model via PockDrug server.

Pockets	Volume Hull	Hydrop-hobic Kyte	Polar Residues Proportion	Aromatic Residues Proportion	Otyr Atom	Number of Pocket Residues	Druggability Probability	Standard deviation
**1**	513.66	-0.87	0.58	0.25	0.0	12.0	0.42	0.1
**2**	313.29	-0.79	0.55	0.18	0.0	11.0	0.36	0.04
